# Effects of Medicinal Plants and Phytochemicals in Nrf2 Pathways during Inflammatory Bowel Diseases and Related Colorectal Cancer: A Comprehensive Review

**DOI:** 10.3390/metabo13020243

**Published:** 2023-02-07

**Authors:** Lucas Fornari Laurindo, Mariana Canevari de Maio, Giulia Minniti, Natália de Góes Corrêa, Sandra Maria Barbalho, Karina Quesada, Elen Landgraf Guiguer, Kátia Portero Sloan, Claudia R. P. Detregiachi, Adriano Cressoni Araújo, Ricardo de Alvares Goulart

**Affiliations:** 1 Department of Biochemistry and Pharmacology, School of Medicine, University of Marília (UNIMAR), Avenida Hygino Muzzy Filho, 1001, Marília 17525-902, São Paulo, Brazil; 2 Department of Biochemistry and Pharmacology, School of Medicine, Faculdade de Medicina de Marília (FAMEMA), Avenida Monte Carmelo, 800, Marília 17519-030, São Paulo, Brazil; 3 Postgraduate Program in Structural and Functional Interactions in Rehabilitation, University of Marília (UNIMAR), Avenida Hygino Muzzy Filho, 1001, Marília 17525-902, São Paulo, Brazil; 4 Department of Biochemistry and Nutrition, School of Food and Technology of Marília (FATEC), Avenida Castro Alves, 62, Marília 17500-000, São Paulo, Brazil; 5 Texas Institute for Kidney and Endocrine Disorders, Lufkin, TX 75904, USA

**Keywords:** inflammatory bowel disease, Crohn’s disease, ulcerative colitis, medicinal plants, phytochemicals, inflammation, oxidative stress, apoptosis

## Abstract

Inflammatory bowel diseases (IBDs) are related to nuclear factor erythroid 2-related factor 2 (Nrf2) dysregulation. In vitro and in vivo studies using phytocompounds as modulators of the Nrf2 signaling in IBD have already been published. However, no existing review emphasizes the whole scenario for the potential of plants and phytocompounds as regulators of Nrf2 in IBD models and colitis-associated colorectal carcinogenesis. For these reasons, this study aimed to build a review that could fill this void. The PubMed, EMBASE, COCHRANE, and Google Scholar databases were searched. The literature review showed that medicinal plants and phytochemicals regulated the Nrf2 on IBD and IBD-associated colorectal cancer by amplifying the expression of the Nrf2-mediated phase II detoxifying enzymes and diminishing NF-κB-related inflammation. These effects improve the bowel environment, mucosal barrier, colon, and crypt disruption, reduce ulceration and microbial translocation, and consequently, reduce the disease activity index (DAI). Moreover, the modulation of Nrf2 can regulate various genes involved in cellular redox, protein degradation, DNA repair, xenobiotic metabolism, and apoptosis, contributing to the prevention of colorectal cancer.

## 1. Introduction

For thousands of years, several plants have been used as the primary source of medicine. Many current drugs for typical disease treatment have originated from medicinal plants, such as metformin, used against diabetes, even though new drugs have been developed using synthetic chemistry. Plant and plant-derived phytochemicals have been considered due to their therapeutic applications, fewer side effects, and low costs in several conditions, such as inflammatory and immunomodulated diseases [1,2,3,4]. Currently, the clinical treatment of inflammatory bowel diseases (IBD) is mainly based on synthetic drugs and surgery that cause serious adverse effects and have questionable effectiveness [5].

Therapeutically, IBDs involve incurable diseases, and their treatments focus on inducing and maintaining remission periods, reducing the necessity of patients’ hospitalization and surgery. IBDs are recurrent and remissive gastrointestinal illnesses affecting approximately 0.3% of Western populations, with a peak onset between the ages of 20 and 30. Crohn’s disease (CD) and ulcerative colitis (UC) are the most typical representatives. Although IBD patients usually have gastrointestinal symptoms, CD and UC can be distinguished from each other using different pathological features, including histopathological analyses and physical and colonoscopy examinations. Signs and symptoms of systemic inflammation may also occur, resulting in extraintestinal manifestations [4,6,7].

Pathologically, the causes of IBDs are still not completely understood. Many authors highlight numerous genetic and environmental factors, including more than 100 genes and smoking, inadequate nutrition, or stress [8,9]. Among the included genetic factors, genes involved in regulating oxidative stress (OS), redox signaling, inflammation, and electrophilic stress are mainly studied [10]. A master regulator of the aforementioned is the Nuclear Factor Erythroid 2-related factor 2 (Nrf2), which is speculated to have protective effects on IBD. Nrf2 is sad to be a key transcription factor that regulates cellular homeostasis in response to, principally, OS. When localized in the cell’s cytoplasm, Nrf2 is suppressed by the Kelch-like ECH-associated protein 1 (Keap1), which is a substrate adaptor protein. Under stress conditions, the cysteine thiols of Keap1 molecules are modified, thereby preventing the degradation of Nrf2 and permitting its translocation from the cytosol to the cell’s nucleus. Nrf2 binds to its target-specific genes inside the nucleus, which mainly encodes antioxidant and detoxification enzymes [11]. Nrf2 also controls metabolic reprogramming, proteostasis, unfolded protein response, mitochondrial biogenesis, autophagy, and immunity, so its role in IBDs might be stronger than was imagined [12].

The unphysiological and sustained chronic inflammation environment promoted by IBD intestinal mucosal inflammation is also the major risk factor for the development of colitis-associated colorectal cancer. Colorectal-associated colitis carcinogenesis is recognized as the third most deadly cancer worldwide due to its high prevalence among the elderly population. It shares many molecular similarities with sporadic colorectal cancer, and preclinical investigators have demonstrated the potential role of Nrf2/Nuclear Factor kB (NF-κB) alterations even in the first stages of cancer occurrence [10,13,14].

**Figure 1:**
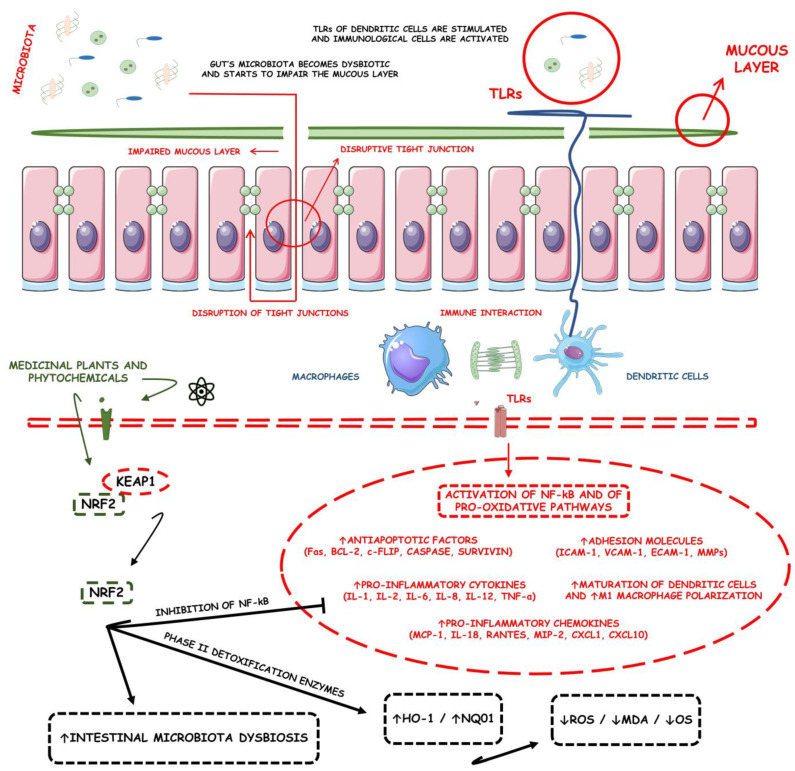
Inflammatory bowel diseases and regulation of Nrf2 using medicinal plants and phytochemicals. ↑, increase; ↓, decrease; Bcl-2, B-cell lymphoma 2; c-FLIP, cellular FLICE-inhibitory protein; CXCL1, chemokine (C-X-C motif) ligand 1; CXCL10, chemokine (C-X-C motif) ligand 10; ECAM-1, ECAM-1, extracellular cell adhesion molecule 1; ICAM-1, intercellular adhesion molecule 1; IL-1, interleukin 1; IL-2, interleukin 2; IL-6, interleukin 6; IL-8, interleukin 8; IL-12, interleukin 12; IL-18, interleukin 18; KEAP1, Kelch-like ECH Associated Protein 1; MCP-1, monocyte chemoattractant protein-1; MIP-1, macrophage inflammatory protein 1; MMPs, metalloproteinases; NF-kB, nuclear factor kappa b; Nrf2, nuclear factor erythroid 2-related factor 2; RANTES, regulated upon activation, normal T cell expressed and secreted; TLR, toll-like receptor; TNF-α, tumor factor necrosis alfa; VCAM-1, vascular cell adhesion protein 1.

Many different plants and phytochemicals have long been recognized to ameliorate inflammation and OS through Nrf2 signaling activation (Figure 1). Accordingly, the plant kingdom can pave the road to the construction of a successful treatment for CD and UC. Many in vitro and in vivo studies using phytocompounds as modulators of the Nrf2 signaling in IBD have already been published [15,16]. However, no existing review emphasizes the whole scenario for the potential of plants and phytocompounds as regulators of Nrf2 in IBD models and colitis-associated colorectal carcinogenesis. For these reasons, this study aimed to build a review that could fill this void. The included studies regarding medicinal plants and natural and synthesized phytochemicals as regulators of the Nrf2 pathways in IBD and colitis-associated colorectal carcinogenesis models can be seen in Table 1 and Table 2, respectively.

## 2. Physiopathology of CD

### 2.1. Definition and General Aspects

CD is a severe chronic inflammatory disease of the gastrointestinal tract (GIT) with relapsing–remitting behavior. It is also called regional ileitis due to its frequent involvement of the ileum, which can occur anywhere in the GIT, being typically transmural. Its etiology is still unknown, but studies defend a multifactorial environmental, immunological, and genetic interaction that contributes to uncontrolled mucosal inflammation. The pathogenesis of CD involves changes in the intestinal microbiota, dysfunctions in the epithelium, and immune hyperreactivity, generating an uncontrolled inflammatory state due to dysregulation of the intestinal epithelial immune response, with the stimulation of pro-inflammatory and inhibition of regulatory pathways. Genetic factors play an essential role, as the concordance rate for monozygotic twins reaches 50%, contributing to the disease’s phenotypic expression. There are more than 160 genes associated with IBDs; many are shared between CD and UC. Several genes associated with IBD overlap with genes involved in the response against mycobacteria, supporting the idea that host interactions with such microorganisms are essential in the pathophysiology of CD. Environmental factors such as pathogenic microorganisms, microbiota, diet, smoking, and psychosocial factors also play an important role in its pathogenesis. Thus, improving life habits could change the microbiome to eubiosis, contributing to the stabilization of the disease [81,82,83,84,85,86,87,88,89].

### 2.2. Genetics Influencing the Pathogenesis of CD

The genes involved in CD may be involved in three stages of inflammation, namely (1) the recognition of pathogens, (2) the elimination of pathogens via innate and cellular immunity, and (3) the prevention of the invasion of pathogens through the intestinal barrier. Therefore, in comparisons of single nucleotide polymorphisms (SNPs) in patients with and without IBDs, there are abnormalities mainly in the functions of modulators of immune function, autophagy, and epithelial function that participate in the interaction between the host and microorganism [85,89].

The gene most associated with CD is nucleotide oligomerization binding domain 2 (NOD2). It encodes an innate immune intracellular protein involved in recognizing bacteria, binding to bacterial peptidoglycans, and subsequent stimulation of the immune system, activating signaling cascades, including the NF-κB pathway, expressed mainly by immune cells and antigen-presenting cells (APCs). As a result, pro-inflammatory and antimicrobial molecules such as interleukin (IL)1-β, IL6, IL8, tumor factor necrosis alfa (TNF-α), and α-defensins are released, which recruit innate immune cells with subsequent activation of adaptive immunity. Furthermore, NOD2 is an essential regulator of cell proliferation, differentiation, and apoptosis mediated by the mitogen-activated protein kinase (MAPK) pathway. Mutations in this gene impair the recognition of invading pathogens by Paneth cells, causing inflammatory intestinal lesions and the lower release of antimicrobial proteins, such as α-defensin. These mutations are probably related to a weakened immune response due to lower activation of the NF-κB transcription factor. However, even with the increased risk associated with NOD2 polymorphisms, only 10% of people with such variants will develop the disease. Finally, NOD2 is central to bacterial autophagy, which initiates by recruiting the molecular autophagy ATG16L1 (type-16 related to autophagy) to the bacterial uptake site. The mutation in ATG16L1 increases the risk of developing IBDs by impairing the ability of autophagy in Paneth cells in cases of intracellular pathogen degradation, indirectly affecting their ability to secrete antimicrobial products, which favors bacterial proliferation and invasion. The ER stress of specialized intestinal epithelial cells (IECs) can be induced by various external factors, initiate a pathological unfolded protein response (UPR), and thus trigger intestinal inflammation. ATG16L1 polymorphisms in IECs determine the tolerable level of ER stress. It defines the activation of the UPR sensor inositol-requiring enzyme 1α (IRE1α), and the hyperactivation of this enzyme spontaneously generates ileitis in mice. Furthermore, signal transducer and activator of transcription 3 (STAT3) and NF-κβ signaling in IECs are also relevant since defects in these two pathways favor the formation of colitis. The NF-κβ kinase-α inhibitor (IKKα) phosphorylates ATG16L1 and thus prevents its degradation. Without this inhibitor, IRE1α accumulates and relays a pathological UPR. The IRGM gene (immunity-related GTPase M), which encodes guanosine triphosphate M, in addition to LRRK2, which encodes leucine-rich repeat protein kinase 2, when mutated, increases the risk of CD. Patients with CD with LRRK2 mutation have increased activation of intestinal dendritic cells, with the exaggerated release of pro-inflammatory molecules. The non-receptor tyrosine phosphatase type 2 (PTPN2) is an autophagy-related gene expressed by lymphoid and Paneth cells and causes the defective formation of autophagosomes. Its loss-of-function mutation in T cells increases the differentiation of T helper (TH) TH0 cells into TH1 and TH17 and reduces regulatory T cells. Therefore, the genes above manifest their polymorphisms as abnormalities in the activity of Paneth cells, which are IECs at the base of Lieberkühn’s crypts in the small intestine. The Cadherin 1 (CDH1) gene is susceptible to IBDs, thus increasing risks for CD. Its function encodes the epithelial protein E-cadherin, which is necessary for tight intestinal junctions. Mutations in this gene generate a more permeable intestinal wall, facilitating the invasion of pathogenic microbes. The HNF4-α gene encodes the hepatocyte nuclear factor 4-alpha, which modulates the expression of genes related to epithelial proliferation and tight junction formation, in addition to regulating adhesion and junction proteins, and, in its absence, malformation of the intestinal wall occurs. Human leukocyte antigen (HLA) genes play a role in the presentation of antigens to effector T cells and may be related to the acquisition of immunological tolerance. Specific mutations in its structure can generate greater risk, while others can act as protectors for CD [81,82,86,87,89].

### 2.3. Immune Response in CD

Several factors reinforce the role of mucosal immune responses in the pathogenesis of CD. Innate and adaptive immunities are the primary controllers of CD pathogenesis. The inflammatory process seems to be caused mainly by the failure of innate recognition of several bacteria, by the impaired intestinal barrier integrity, and by the spread of luminal pathogens through the intestinal wall, as previously mentioned, overloading innate immune cells to recognize and eliminate invaders [81,84,89].

Cells such as neutrophils, macrophages, innate lymphoid cells (ILCs), monocytes, dendritic cells, and Paneth cells make up the intestinal innate immune response. Neutrophils, in a balanced environment, phagocytose pathogenic microorganisms. Still, when there is accumulation in the intestinal epithelium, it ends up compromising the intestinal epithelial barrier, stimulating the production of substances that result in inflammation. By phagocytosing microorganisms, they mainly fight extracellular bacteria and fungi, in addition to forming extracellular neutrophil traps (NETs), which immobilize large pathogens and allow localized lysis. Due to the digestive action of neutrophils, the damage may even be greater than the initial insult for which it had been recruited. In addition, the antimicrobial defense of neutrophils is defective in patients with CD, with impaired nicotinamide adenine dinucleotide phosphate (NADPH) oxidase activity of these cells and reduced inflammatory response against dead *Escherichia coli* compared to patients with UC. Macrophages in a healthy intestinal mucosa control tissue remodeling by clearing apoptotic or senescent cells, and the alteration in their phagocytic function may contribute to the pathogenesis of CD. Studies have shown that such cells were significantly affected in CD patients, with reduced secretion of protective substances, such as IL-10, and increased synthesis of inflammatory molecules, generating a late elimination of microbial agents with an exacerbated inflammatory response. On the other hand, ILCs produce cytokines responsible for communication between the innate and adaptive immune systems. In patients with CD, in the inflamed ileum and colon, there is an increase in ILC1 and ILC3. Furthermore, areas with inflammation have high levels of interferon (IFN)-γ-producing ILC1s at the expense of ILC3, which synthesizes the anti-inflammatory cytokines IL-17 and IL-22, which suggests the increased transformation of ILC3 to ILC1, mediated by the inflammatory cytokine IL-12, may contribute to the disease [87,89].

Monocytes perform phagocytosis, respond to damage-associated molecular patterns (DAMPs) through toll-like receptor (TLR) signals, are tumor factor necrosis (TNF) producers, and stimulate mucosal inflammation, increasing tissue damage. In acute inflammation, monocytes differentiate into mature macrophages. In homeostasis, IECs produce transforming growth factor beta (TGF-β), thus increasing the synthesis of the anti-inflammatory cytokine IL-10 by dendritic cells. In inflammatory states, these cells express toll-like receptor 2 (TLR2) and TLR4, which stimulate the production of pro-inflammatory cytokines in scenarios of frequent interaction with the intestinal microbiota. They also promote cell activation and differentiation of TH0 cells into TH1, TH2, and TH17 and induce other essential inflammatory cells, such as natural killer cells. TH cells are activated and exert a TH1-like response. Most lymphocytes are activated in the intestinal lymphoid tissue and then go to the site of inflammation. Integrins, present on the leukocyte surface, are essential for leukocyte extravasation, as they make them bind to cell adhesion molecules present on the endothelial wall. In patients with CD, it is relevant that mucosal effector T cells may be resistant or less responsive to suppression mediated by Treg cells, which contributes to the maintenance of intestinal inflammation [81,87,89,90].

Furthermore, humoral immunity is also relevant, given that altered levels of mucosal and secretory antibodies were detected in patients with CD with considerably elevated IgG levels against bacterial cytoplasmic proteins and reduced secretory IgA. In addition, CD patients often have anti-*Saccharomyces* disruption antibodies (ASCAs). However, the contribution of plasma cells and B cells in the pathogenesis of IBDs has not yet been elucidated [82,89,90].

Thus, in a few words, CD originates due to the exacerbated responses of TH1 and TH17 cells to inflammatory cytokines synthesized by APCs and the release of pro-inflammatory cytokines IL-17, TNF, and IFN-γ, which maintain inflammation by stimulating the synthesis of more inflammatory cytokines, such as IL-1, IL-6, IL-8, IL-12, IL-18, and TNF by other cell types such as endothelial, monocytes, and macrophages. IL-12 and IL-23, produced by innate immune cells such as dendritic cells and macrophages and act as modulators of intestinal inflammation. Given the importance of inflammation in CD, current therapies block the synthesis of inflammatory mediators, stopping the accelerated inflammation [87,89].

## 3. Physiopathology of UC

### 3.1. Definition and General Aspects

UC is defined as a continuous and diffuse inflammatory process that affects the epithelium and intestinal mucosa only of the colon and rectum. The classical clinical presentation is characterized by bloody diarrhea, abdominal pain, fecal urgency, and/or tenesmus. The prevalence of UC increases annually, ranging from 8.8 to 23.1 per 100,000 individuals-year. In North America, the range is 0.6 to 24.3 per 100,000 individuals-year, and in Europe, the range is 7.3 to 17.4 individuals-year. The disease has a principal peak in people in the second and third decades of life, regardless of sex, and a second smaller peak in the sixth and seventh decades of life [91,92,93,94]. The diagnosis is based on clinical symptoms and signs, endoscopic evaluation, and histologic parameters in the absence of demonstrable alternate etiology. Infectious etiology, particularly *Clostridium difficile*, must be ruled out at the time of diagnosis. Nonetheless, the gold standard for diagnosis is ileum colonoscopy with biopsies [95,96].

The objective of treatment is based on mucosal healing, and this is performed using drugs such as mesalamine in uncomplicated patterns of disease, glucocorticoids in acute cases, and, in more complicated forms, azathioprine, biological agents, Janus Kinases (JAK) inhibitors (TNF antibodies, biosimilars, vedolizumab, tofacitinib, and ustekinumab) and calcineurin inhibitors. Proctocolectomy should be considered in refractory cases or high-grade epithelial dysplasia [96,97,98].

### 3.2. Genetics Influencing the Pathogenesis of Ulcerative Colitis

Environmental and host factors influence the susceptibility to the development of UC. Antibiotic use, smoking, air pollution, mental and psychological state, physical exercise, and diet are fundamental points in the pathogenesis of IBDs. The pathology of the disease may start with events that impair the mucosal barrier, cause intestinal microbiota imbalance, and encourage an abnormal intestinal immune response. In addition, genetic predisposition is relevant for the development of the disease [99,100].

Although only 8% to 14% of patients with UC have a family history of IBD, first-degree relatives of people diagnosed with this condition have a 4-fold risk, monozygotic twins have concordance rates of 6% to 13%, and the Ashkenazi Jewish population, when compared to other ethnicities, has an increased risk of 3 to 5 times. It is known that multiple genetic polymorphisms drive the genetic basis of the disease and that genetic studies have found about 260 IBD-associated susceptibility loci [101]. Many of the genes identified are involved in different aspects of intestinal immunity, including intestinal mucosal barrier function, autophagy, epithelial restitution, microbial defense, and regulation of adaptive immunity [91].

### 3.3. Genetics Influencing the Pathogenesis of Ulcerative Colitis

The GIT mucosa is exposed to millions of antigens from food, the environment, and the microbiome. The GIT epithelium is covered with a thick layer of mucin (the first line of defense for the intestinal immune system), which provides a physical separation between antigens and intestinal immune cells but also has antimicrobial properties. In UC, the synthesis and secretion of mucin are impaired, which generates epithelial damage and increases the permeability of the mucosa to luminal pathogens, resulting in increased uptake of these antigens and increased potential for stimulation of the system. Concomitantly, it is believed that colonocytes, colonic epithelial cells, in UC have impaired expression of the peroxisome proliferator-activated receptor g (PPAR g), a nuclear receptor that regulates inflammation negatively [102].

Activation of the innate immune response by antigens occurs through antigen-presenting cells and T cells. In addition, there is also an increase in the activation and sensitivity of mature dendritic cells, helping to generate inflammation. These dendritic cells express an abundance of TLR, which use pathogen pattern recognition to signal the activation of multiple transcription factors, such as the NF-κB transcription factor, which triggers inflammatory cascades. These inflammatory cascades produce pro-inflammatory cytokines, such as TNF-α, IL-12, IL-23, IL-21, and IL-24. These cytokines send signals through intracellular proteins, such as JAK, which further potentiate the activation and proliferation of lymphocytes [103].

Another cell involved in the immune response is neutrophils, which are recruited in abundance with a histology characteristic of “crypt abscesses” in UC. The inflammatory environment of UC promotes neutrophil survival (potentially via hypoxia-inducible factor 1-alpha (HIF-1)). This increased survival increases neutrophils’ inflammatory actions and the consequent tissue damage through reactive oxygen species (ROS) and pro-inflammatory cytokines production. Furthermore, the formation of NETs functions as a reservoir for immunogenic molecules that sustain the inflammatory response. Therefore, it is believed that after disease onset, the previous wave of innate inflammatory neutrophils and monocytes (with their repertoire of pro-inflammatory cytokines, e.g., IL-1, IL-6, and TNF-α family) creates an inflammatory environment. The adaptive immune response of UC occurs after the initial presentation of antigens to CD4 T cells, in which they promote a non-classical Th2 response, with augmented levels of IL-4, IL-5, and IL-13 but mainly IL-4 [104].

## 4. Nrf2 Signaling Pathway

### 4.1. Historical Perspective of Nrf2

The general notion of Nrf2 emerged in the 1970s when investigations into the transcriptional control of its antioxidant response began. From this, several studies started on its anticancer effects due to its ability to increase detoxifying phase II and activating enzymes such as glutathione-S-transferase [105,106].

In 1989, the action of activator protein 1 (AP-1) and erythroid nuclear factor 2 (NF-E2) was described, the latter being identified as the first of the six cap’n’ transcription factors collar, with nuclear respiratory factor (NRF) 1, NRF2, NRF3, BTB and CNC homology (BACH) 1, and BACH2 identified shortly thereafter. The NF-E2/AP-1 sequence was associated with a promoter of the glutathione transferase (GST)-Ya gene, being responsive to t-butyl hydroquinone (t-BHQ), when it resulted in responses to its antioxidant action, giving rise to the term “antioxidant response elements” (AREs). This fact then indicated that the transcription of phase II detoxifying enzymes was sensitive and directly influenced by electrophiles, a key functional feature of most Nrf2 activators. With this idea, it was believed that the redox sensitivity of Nrf2 was conferred by a regulator capable of detecting oxidative/electrophilic stress [107,108,109,110].

The molecular interactions that act on Nrf2 signaling were uncovered in the 1990s when it was first determined that this molecule is downregulated by Keap1. In the 2000s, it was discovered that cysteines interacted with Keap1 in the electrophilic stress pathway and were modulated to different inducers, which became known as the “cysteine code’’ [111].

Until now, Nrf2 was described as a molecule regulating inflammatory metabolism, expressed in several tissues and organs, such as the heart, brain, liver, kidney, and skin. Nrf2 signaling is gaining ground as it covers crucial roles in essential processes in the body, such as embryonic development, OS, and ischemia/reperfusion injury. In addition, there are reports that this molecule regulates free radical deputation and lipid homeostasis [112,113,114,115,116,117].

### 4.2. Nrf2 Regulation, Signaling Pathways, and Repercussions

Epigenetic factors and interaction with several signaling pathways work in the maintenance of Nrf2 levels. In addition to Keap1, the regulation also happens by three other ubiquitin ligase E3 complexes responsible for the ubiquitylation and degradation of Nrf2 including the Cullin 3 (CUL3)-RING-box protein 1 (RBX1)-KEAP1 complex, which is responsible for the response to electrophilic/OS in the cytosol; the Skp, Cullin, F-box containing complex (SCF^/β-TrCP^ complex), which acts both in the nucleus and in the cytosol through metabolic changes; and, finally, the endoplasmic reticulum (ER)-transmembrane E3 ubiquitin ligase (HRD1), which is located in the endoplasmic reticulum and ubiquitylates Nrf2 during stress in this cellular compartment [110,118,119,120,121].

As seen earlier, Keap1 is the main intracellular regulator of Nrf2, which contains 589 amino acids and is a cap ‘n’ collar leucine zipper transcription factor. The latter includes seven functional domains, including the NRF2 ECH homology (Neh) domains Neh1-Neh7, which help regulate and stabilize Nrf2 [114,122]. Under appropriate stimulation by physical and chemical factors, including trauma, fever, and inflammation, which are transcriptional regulators, Nrf2 dissociates from Keap1 in the cytoplasm, which induces ubiquitination and proteasomal degradation of Nrf2 [112,123].

Post-transcriptional processing is involved as another activation mechanism, and the correct functioning of Nrf2 is modifications of the Nrf2 gene (NFE2L2) mRNA transcript. This event occurs from regulation by microRNAs (miRNAs), adenosine methylation, long non-coding RNAs (lncRNAs), and alternative splicing of the NFE2L2 transcript. Thus, miRNAs down-regulate or up-regulate the expression and activity of the Nrf2 protein by directly targeting the 3′-untranslated region (UTR) sequences of NFE2L2 or KEAP1 mRNA, respectively [124,125]. After this process, there is an exposure to chemicals (often electrophiles) or ROS, which allows the activity of the ubiquitin E3 ligase of the Keap1-CUL3 complex to decrease and Nrf2 to stabilize, accumulate in the nucleus, and activate its target genes. At the same time, this molecule is recognized and binds to the core sequence of ARE [112,114]. 

As a consequence of this series of actions, several downstream antioxidants are activated, which generates positive feedback on the expression levels of inflammatory proteins and detoxification enzymes. The transcribed genes involve superoxide dismutase (SOD), glutathione (GSH), glutathione peroxidase (GPX), and heme oxygenase (HO-1), which have been identified to suppress ROS and reduce OS [114,126].

There is growing evidence to suggest that Nrf2 is essential in OS injury, where Nrf2 is involved in several signaling pathways, including NF-κB and other cytokine signaling pathways [127]. Thus, there is a relationship between Nrf2/ARE signaling and several necessary pathophysiological conditions, including hypoxia, ischemia, fibrosis, and apoptosis [128]. Ohkoshi et al. [129] found that mice lacking Nrf2 (Nrf2−/−) are more susceptible to diseases based on OS and drug-induced toxicity. The activation of Nrf2 by chemical inducers or genetic factors, such as disruption of the Keap1 gene, significantly reduces damage caused by OS and prevents the development and exacerbation of stress-induced diseases.

As Nrf2/Keap1 signaling plays a critical role in the maintenance of intracellular redox homeostasis, it also plays a role in a wide range of cells and organs in different types of conditions, such as cardiac diseases related to oxidative damage, including ischemic myocardial disease, heart failure and hypertrophic heart disease [130]. In other pathologies, a lower expression of Nrf2 mRNA was observed in all patients with refractory sensorineural hearing loss before corticosteroid treatment [131]. Yang & Zhang [132] revealed an age-dependent Nrf2 dysfunction, which raised the hypothesis of leading to dementia/vascular cognitive impairment by acting on the cerebrovascular system and neural cells. Against IBDs, many authors suggest that upregulation of Nrf2 is effective in promoting colon protection and intestinal health due to anti-inflammatory and antioxidant effects [11,133,134].

### 4.3. Nrf2 Regulation by Phytochemicals

As seen before, the regulation of Nrf2 depends on many factors, such as genetics, drugs, and the molecular environment. Phytochemicals have been documented as modulators of Nrf2 signaling, reversing hypermethylated states by inhibiting DNA methyltransferases (DNMTs) and histone deacetylases (HDACs) by inducing translocation enzymes or even by inducing miRNA to target the 3′-UTR of the corresponding mRNA transcripts, which contains around 22 nucleotides [124,125,132,135].

Many compounds such as curcumin, sulforaphane, resveratrol, and 3,3′-diindolylmethane can protect cells from inflammation and OS by mediating the Nrf2 defense response and stimulating phase II detoxification enzymes, transporters, and antioxidants, which is effective in preventing further damage [16,124,132,135,136,137,138,139,140].

### 4.4. Nrf2 Signaling Pathway: Implications for IBDs

As aforementioned, Nrf2 corresponds to a stress-responsive transcription factor associated with maintaining cellular homeostasis and, under normal conditions, is kept in the cytoplasm of the cells by Keap1. The primary function of Keap1 is to facilitate the degradation of Nrf2 when it is not necessary. However, when OS achieves normal cells, these events trigger the dissociation between Keap1 and Nrf2, dissolving the Nrf2/Keap1 complex and promoting the translocation of Nrf2 to the cells’ nucleus where Nrf2 induces the activation and transcription of a variety of anti-oxidative and anti-inflammatory genes [10,119,120,121,141].

During the development of gastrointestinal diseases, the Nrf2/Keap1 axis plays a crucial role in maintaining bowel health and proper functionality. It is known that the Nrf2/Keap1 pathway is not only directly involved in the prevention of IBDs but also in the prevention of CD- and UC-related complications, such as intestinal fibrosis and colorectal cancer. Although the roles of Nrf2/Keap1 in the development of UC are still elusive, this complex’s functions in CD are better described. Most studies concentrate on the abilities of the complex to modulate the production of many pro-inflammatory cytokines (such as IL-6, IL-17, and IL-1β), controlling the levels of antioxidant enzymes (such as HO-1 and NAD(P)H quinone oxidoreductase-1 (NQO1)) and extracellular matrix (ECM) degradation proteins (metalloproteinases (MMPs)) and modulating intestinal cells’ autophagy [10]. However, Gerstgrasser et al. [142] conducted a study on genetically modified mice constitutively expressing active Nrf2 and found that all genetic activation of the Nrf2/Keap1 axis either on epithelial or in myeloid cells could aggravate acutely, but not chronically, the mucosal inflammatory processes. Upon acute mucosal damage, tighter regulation of the redox balance and excessive ROS from an over-induction of antioxidant enzymes can be detrimental to the mucosal ambiance. By contrast, Zhao et al. [143] reported that Nrf2 deficiency increases oxidative damage and induces Th17 cell differentiation, which is, by nature, pro-inflammatory cells.

Inflammation results in the production of ROS, which are cardinal events in the physiopathology of IBDs and lead to significant activation of Nrf2. Nrf2′s control of the inflammatory mediator production relies principally on the fact that Nrf2 possesses immunomodulatory functions, which affect the Nrf2-Keap1 pathway and protect host cells from inflammatory disorders. However, the tasks of the Nrf2-Keap1 axis are not always unidirectional for protection but depend on the type of immune cell targeted. As an example, the activation of Nrf2 in macrophages suppresses the activation of pro-inflammatory genes in these cells, which decreases the production of ROS and TNF-α, in addition to reducing the expression of TLR4 and the Forkhead box protein O1 (FOXO1) genes, which also decreases the production of IL-1β, IFN-β, and IL-6 and subsequent tissue injury. From another perspective, the disruption of Keap1 in myeloid cells was found to increase the bacterial phagocytic activity of peritoneal macrophages. On the other hand, Nrf2-deficient peritoneal neutrophils were found to increase the expression of IL-6, monocyte chemoattractant protein-1, TNF-α, and macrophage inflammatory protein-2 [11]. Additionally, activation of Nrf2 signaling impairs Th1-induced inflammation and biases T cells towards a Th2 differentiation and an anti-inflammatory response [11,144]. In addition, Nrf2 activation in T CD4 cells promotes and enhances Treg cell activation and expansion. In turn, Nrf2 activation in myeloid-derived suppressor cells (MDSCs) also enhances their expansion, suppressing and inhibiting T-cell responses [11,145,146].

The Nrf2-Keap1 axis also causes effects in a cluster of differentiation 8 (CD8+) antigen-specific T cells. Sha et al. [147] reported that the loss of Nrf2 in bone marrow-derived macrophages impairs antigen-driven CD8+ T cell function by limiting GSH and Cys availability. The Nrf2 signaling pathway also affects dendritic cells by suppressing their functions and facilitating the immune escape of antigens. The Nrf2 inhibition of dendritic cells, in turn, promotes T-cell proliferation and its differentiation into Th1 [148]. Theoretically, the exposure of the immunological system to OS is the main reason for the increases in inflammatory disease activities. Nrf2 senses OS, and this factor induces an intracellular signaling pathway in the immune cells to ideally trigger and mediate the innate immune response, which is followed by the differentiation of the adaptative immune cells. The fact is that a severe OS state can be much more than physiologically compensated by the activation of the Nrf2 response. In this case, Nrf2 up-regulation increases and does not compensate the immunological response, leading to excessive inflammation. So, although the Nrf2 agonism during inflammatory and oxidative diseases is fundamental to restoring homeostasis, the Nrf2 decompensation causes excessive inflammation and turns the condition into an aggravation process led by inflammation [11].

However, Nrf2 inhibits NF-κB activity via different mechanisms. During the development of IBDs, the activation of inflammatory pathways is fundamental and results in the production of many pro-inflammatory cytokines and other pro-inflammatory mediators, such as IFN-γ, TNF-α, IL-1β, and IL-12. These changes in IBDs are related mainly to the activation of the NF-κB inflammatory signaling associated with an exacerbated immunological response against the increased pathogenic bacteria concerning commensals through immune cells such as macrophages, monocytes, lymphocytes, and epithelial cells of the intestinal lymphoid tissue. NF-κB is central to inflammatory responses, and its expression depends on the redox status. When the levels of ROS and reactive nitrogen species (RNS) are elevated, these molecules stimulate and exacerbate the inflammation that is mechanistically linked to the p65 subunit of the NF-κB signaling pathway. During quiescence, NF-κB remains inactivated physically due to the direct interaction with its inhibitor, IkBα, which prevents the nuclear localization of NF-κB and its further stimulative actions on pro-inflammatory genes expression. However, during expressive redox disruption stimuli, IkB kinase (IKKβ) is activated and phosphorylates IkBα, which is then targeted for degradation. With the IkBα degradation, this inhibitor is removed from NF-κB and translates the factor to the cell’s nucleus. Once inside the nucleus and in contact with the gene expression machinery, NF-κB can modulate and exacerbate inflammation [11].

The interplay between Nrf2 and NF-κB is a complex game in which many aspects of their relationship must be analyzed. Firstly, it is known that IKKβ possesses an ETGE motif that can bind Keap1 to target it for ubiquitination and further proteasomal degradation. Thus, under conditions of massive oxidation, such as during ROS abundance, Keap1 is inhibited, thereby signalizing to IKKβ, which in turn leads to the phosphorylation of IκBα and its further degradation. These events may result in an aberrant induction of NF-κB activation and, thus, in a massive expression of pro-inflammatory stimuli. Nevertheless, NF-κB transcriptionally activates at a specific kB promoter site the expression of Nrf2 signaling, thus tenting against its activation. This happens due to the competition between NF-κB and Nrf2 for the same coactivator histone acetyltransferase CREB-binding protein (CBP)/p300. In this competition, NF-κB, when up-regulated, may disrupt the interaction between the Nrf2 and the CBP’s CH1-KIX domains, thus decreasing Nrf2 activation [11].

In addition, NF-κB also promotes the recruitment of many corepressor histone deacetylases 3 (HDAC3) in an attempt to induce the Nrf2-driven expression of ARE [149]. However, at this same time, NF-κB signaling inhibits the Nrf2-ARE pathway through an interaction between Keap1 and p65. This happens due to the NF-κB binding to Keap1 and further translocation of Keap1 to the cell’s nucleus, favoring Nrf2′s ubiquitination and degradation [150].

Similarly, Nrf2 inhibits the most important inflammatory factor in IBDs, the NF-κB, through several different cellular and biochemical mechanisms. Chronically, it is known that NF-κB activity is inhibited when Nrf2 expression is the most active. Nrf2 primarily inhibits NF-κB by suppressing ROS. Nrf2 activators reduce ROS presence and inhibit the ROS-mediated activation of the NF-κB-dependent production of pro-inflammatory factors [151]. In concordance with the data mentioned above, cells with deficient Nrf2 signaling showed increased expression of the p65- NF-κB protein, although mRNA cellular levels remained unchanged [152]. Lipopolysaccharides (LPSs) are also great stimulators for the expression of NF-κB pro-inflammatory signaling, activating faster NF-κB response and slower Nrf2 activation. It is known that the induction of pro-inflammatory cytokines production by LPS stimuli through the activation of the TLR4/NF-κB axis promotes the relative expression of Nrf2, followed by its necessary translocation to the cells’ nucleus. However, Nrf2 strikingly inhibits the expression of many pro-inflammatory cytokines such as IL-1β, IL-6, and TNF-α by only binding to the p65 molecules directly [11,153]. Because of that, Nrf2 controls the pro-inflammatory polarization of different immune cells, such as in the case of M1-polarized macrophages, which are inhibited by the Nrf2-derived blocked transcriptions of pro-inflammatory mediators (TNF-α, IL-6, and IL-1), which are typically produced by pro-inflammatory macrophages and are necessary for the proper functioning of these cells [154]. Additionally, studies regarding the activation of Nrf2 signaling among macrophages have reported that activation of Nrf2-derived metabolites such as itaconate could not only effectively inhibit the NF-κB signaling in macrophages but also promote the macrophages’ M2 anti-inflammatory polarization [155]. Nrf2 anti-inflammatory effects were also highlighted in neutrophils. It was found that in Nrf-knockdown peritoneal neutrophils, the LPS-induced elevation of monocyte chemoattractant protein-1 (MCP-1), IL-6, TNF-α, and other pro-inflammatory mediators were more pronounced along with increased ROS levels when compared with neutrophils with perfect Nrf2 signaling [156]. Taken together, it is conclusive that Nrf2 is a negative regulator of LPS-TLR4-induced signaling during many stages of the innate immune responses taken by neutrophils and macrophages, which is mainly implicated during the development of IBDs. 

Specifically, Nrf2 also beneficially affects cyclooxygenase (COX) and inducible nitric oxide (NO) synthase (iNOS) signaling in immunological and inflammatory cells. iNOS corresponds to a pro-inflammatory marker expressed mainly by immunological cells such as T cells, macrophages, and mature dendritic cells. COXs, especially COX-2, are critical enzymes in the inflammatory processes that catalyze the formation of prostaglandins and thromboxane during the arachidonic acid pathway. COX-2 is expressed mainly by macrophages and is up-regulated in response to various growth factors and other inflammatory stimuli, such as in the case of LPSs [11,81]. It was observed that in Nrf2-deficient mice, a significant increase in the expression of COX-2, iNOS, TNF-α, and IL-6, as well as in OS parameters, occurred [157]. Additionally, peritoneal macrophages pretreated with sulforaphane did not respond to LPS stimulation, which potently reduced the expression of TNF-α, iNOS mRNA, COX-2, and IL-1β pro-inflammatory mediators by these cells [158].

The levels of proteases such as MMPs, which are zinc-dependent proteolytic enzymes responsible for the occurrence of the extracellular matrix components degradation, antiproteases, and OS parameters, are interconnected. They are also crucial in the Nrf2-mediated regulation of inflammatory processes [81]. Nrf2 overexpression was sufficient to reduce cartilage degradation in a murine model of osteoarthritis due to decreases in MMP-13 expression and reductions in the TNF-α, IL-1β, and IL-6 serum levels [159]. The induction of Nrf2 expression by flavonoids was also associated with decreased cataract formation due to the inhibition of MMP-9 expression and release [160]. Cancer is also an inflammatory condition dependent on MMPs infiltrating healthy tissues during metastasis. Through the Nrf2/HO-1 axis, the expression of MMPs such as the MMP-9 was inhibited and, therefore, the metastasis processes of many cancer cell lines, such as the breast cancer cells [11,161].

As a transcription factor, Nrf2 regulates various genes involved in cellular redox homeostasis and protein degradation, DNA repair, and xenobiotic metabolism. Due to these reasons, the complex role of Nrf2 signaling in cancer evolves continuously since Nrf2 itself is responsible for the mechanisms that preserve cell function and viability during stress. However, the mode of Nrf2 activation often dictates its role in either preventing cancer development or promoting cancer progression. The wide array of cytoprotective target genes controlled by Nrf2 is fundamental in controlling and preventing oxidative, proteotoxic, and metabolic stresses that may contribute to cell malignant transformation. While Nrf2 activation plays a key role in chemoprevention, it was evidenced that prolonged Nrf2 activation can promote cancer initiation and progression as Nrf2 signaling is up-regulated during the first stages of tumor formation. Although this can be just a secondary effect of increased stress associated with the cell malignant transformation, whether Nrf2 is involved in cancer development remains unclear. However, it is known that prolonged Nrf2 activation associates highly with suppressed apoptosis of newly transformed cells by continuous up-regulation and activation of detoxification and DNA repairments. In addition, it stands to reason that the cell malignant transformation itself may not involve Nrf2. Still, the up-regulation of this signaling pathway is involved in mitigating the stress generated by replication errors, which helps mediate tumor growth and survival, as well as cancer initiation and also cancer progression [162,163,164].

Colonic tumorigenesis occurs more frequently in males than in females due principally to lifestyle habits. In colon tumors, Nrf2 plays differential roles in the stages of tumorigenesis. Many studies regarding the purpose of investigating the roles of Nrf2 in colitis-associated tumorigenesis were published in the global scientific literature. Nonetheless, the majority of these studies are in vitro or in vivo and not clinical. C.-H. Song et al. [165] conducted an in vivo study with a dextran sulfate sodium (DSS)-treated mice model of colorectal cancer formation to evaluate the roles of Nrf2 in this cancer development. The results showed that Nrf2 knockout suppressed aggressive colorectal cancer formation development in DSS-induced animals. In addition, the mRNA and protein expression levels of Nrf2-related antioxidants (HO-1 and glutamate-cysteine ligase catalytic subunit) and NF-κB-related mediators (COX-2 and iNOS) were significantly lower in the animal group that possessed Nrf2 knockout than in the wild-type mice group. The expression levels of the 15-hydroxyprostaglandin dehydrogenase (15-PGDH) tumor suppressor were also higher in the Nrf2-knockout group than in the wild-type, which only supports the oncogenic effects of Nrf2 in the later stages of colonic carcinogenesis. In another study, Tin Oo Khor et al. [166] proposed an increased susceptibility of Nrf2-knockout mice to colitis-associated colorectal carcinogenesis while studying DSS-induced colitis. The authors found that Nrf2-knockout DSS-treated mice had increased incidence, multiplicity, and size of all colorectal tumors versus DSS-treated wild-type mice. In addition, the authors also proposed that the proportion of adenocarcinoma tumors was much higher in Nrf2-knockout than in wild-type animals. Biochemically, the knockout mice had increased markers of inflammation in the tumorigenesis tissues, such as COX-2, lipoxygenases, PGE2, and leukotriene B_4,_ compared with wild-type mice. Finally, as expected, the knockout mice did not present elevated phase II detoxifying/antioxidant enzymes NQO1 and UDP-glucuronosyltransferase 1A1.

## 5. Medicinal Plants That Target Nrf2 Pathways

### 5.1. Cynara cardunculus L.

Speciale, Muscarà, et al. [17] worked on an in vitro study with a TNF-α-induced intestinal human colorectal adenocarcinoma (Caco-2) epithelial cells model of colitis to evaluate the effects of *Cynara Cardunculus* leaf extract against Nrf2 deactivation. The Caco-2 cells presented elevated NF-κB activation and ROS intracellular levels and decreased TAA and GSH levels. The plant extract was rich in chlorogenic acid and luteolin, and the results showed that the extract’s use could increase Nrf2 expression via increases in the Nrf2 nuclear levels, glutamate—cysteine ligase catalytic subunit (GCLC) mRNA, and NQO1 mRNA.

### 5.2. Panax ginseng

S. Yang et al., 2022 [18] worked with *Panax ginseng* root extract rich in ginsenosides to evaluate its effectiveness in activating Nrf2 signaling to reduce inflammatory and oxidative stimulation in both an in vitro and an in vivo model. The models presented high levels of NO, TNF-α, IL-6, IL-1β, and ROS, in addition to augmented mitochondrial dysfunction. The results showed that the extract in different concentrations increased the Nrf2 nuclear translocation due to increased p62 phosphorylation and protein kinase b (Akt)-mammalian target of rapamycin (mTOR) signaling.

### 5.3. Rose odorata sweet var. gigantean

Li et al., 2020 [19] evaluated the roles of *Rose odorata sweet var. gigantean* (Coll. Et Hemsl.) Rehd. Et Wils root extract (ROE) in activating Nrf2 signaling using a DSS-induced mice model of colitis. Due to the DSS administration, the mice presented elevations in the disease activity index (DAI), OS (increased NOS, malonaldehyde (MDA), and myeloperoxidase (MPO) levels), loss of epithelial and goblet cells, crypt aberrations, and prominent transmural inflammatory cells infiltration (increased TNF-α, IL-6, and IL-1β). After treatment with different ROE doses, the mice presented elevations in Nrf2 signaling due to principally increased HO-1 levels, as well as increased SOD and decreased numbers of nuclear NF-κB-p65 positive cells, p-NF-κB-p65, p-IKKα/β, and Keap1.

### 5.4. Ficus pandurata Hance

Dai et al., 2021 [20] used a mice model of colitis to evaluate whether the crude extract of *Ficus pandurata* Hance. could significantly ameliorate the disease process. The mice presented increased DAI and augmented intestinal inflammation and OS parameters through increased NF-κB activation and MDA levels and decreased intestinal barrier integrity, diamine oxidase activity (DAO), SOD, and glutathione peroxidase (GSH-Px). After the treatment, the mice presented elevated Nrf2 activation through diminished TLR4/ Innate Immune Signal Transduction Adaptor (MyD88) /NF-κB activation and Keap1, cytochrome b(558) subunit beta or NADPH (nicotinamide adenine dinucleotide phosphate hydrogen) oxidase 2 (NOX-2), and human neutrophil cytochrome b light chain (p22-phox) levels and augmented DAO, total SOD (T-SOD), HO-1, and NQO1 levels and Nrf2 translocation.

### 5.5. Moringa oleifera Lam

Kim et al., 2017 [21], during an in vivo study with a model of colitis, evaluated the effects of an isothiocyanate-enriched *Moringa oleifera* Lam. seed extract on the Nrf2 signaling pathway activation. The mice presented increased DAI and intestinal inflammation (increased TNF-α, IL-1β, IL-6, NO, and iNOS) and OS (increased MPO), as well as decreased intestinal integrity. During the experiment, Nrf2 signaling increased due to decreased pro-inflammatory gene expression, resulting in decreased NF-κB-dependent pro-inflammatory signals. Additionally, the mice presented elevated expression of the transcription factor STP1, NQO1, and HO-1.

### 5.6. Aucklandia lappa Decne

The in vivo and in vitro study by Chen et al., 2022 [22] investigated the role of an aqueous and a sesquiterpene lactones-rich extract fraction from *Aucklandia lappa* Decne in colitis. The studied mice presented an elevated disease activity index (DAI). Both models expressed increases in TNF-α, IL-1β, and IL-6 levels, M1 macrophage polarization, iNOS, and COX-2 levels, NF-κB-related proteins expression, and p38 MAPK, NF-κB p65, extracellular signal-regulated kinases (ERK), and JNK phosphorylation, as well as decreased heme oxygenase 1 gene (HMOX-1) expression. After treatment, the models presented decreases in IL-1β mRNA, IL-6 mRNA, and TNF-α mRNA expression, MAPK, and NF-κB activation, and increased HMOX-1 mRNA, and ↑Nrf2 mRNA expression in the mice and decreased p38MAPK, p-NF-κB-p65, ERK, and JNK phosphorylation and increased Nrf2 and HMOX-1 expression in the cells.

### 5.7. Vaccinium myrtillus and Ribes nigrum

Speciale, Bashllari, et al., 2022 [23] conducted an in vitro study with Caco-2 human intestinal epithelial cells exposed to TNF-α as a model of intestinal inflammation. The cells presented elevated NF-κB activation measured by the TNF-α-induced nuclear translocation of NF-κB-p65 and increased expression of pro-inflammatory cytokines such as IL-8 and IL-6. After treatment with anthocyanins-rich purified and standardized *Vaccinium myrtillus* and *Ribes nigrum* extract, the cells presented diminished NF-κB-p65 translocation and overactivation of the Nrf2 pathways measured by increased Nrf2 nuclear translocation and NQO1 mRNA expression.

### 5.8. Mesua assamica (King&Prain) Kosterm

Puppala et al., 2022 [24] evaluated the effects of the bark ethanolic extract from *Mesua assamica* in NF-κB-RE-luc2P HEK 293 TNF-α-stimulated cells and a DSS-induced C57BL/6 mice model of colitis. The treated cells presented elevated intestinal inflammation and OS. In turn, the animals presented elevated DAI and splenomegaly, as well as reduced intestinal integrity and colon length. The models showed elevated levels of MPO, nitrite, IL-6, IL-1β, and TNF-α and reduced levels of GSH. After treatment with the plant, Nrf2 signaling was activated due to diminished NF-kB-related proteins expression, STAT3 signaling, and IKBα phosphorylation, as well as elevated SOD2 and HO-1 levels and NAD-dependent deacetylase sirtuin-1 (SIRT1) expression.

### 5.9. Vaccinium myrtillus

Zhang et al., 2022 [25] evaluated the roles of *Vaccinium myrtillus* berry anthocyanin extracts in a DSS-induced *Drosophila melanogaster* model of intestinal inflammatory damage. Before treatment, the flies presented diminished intestinal integrity and length and elevated levels of ROS and pro-inflammatory cytokines. The signals of inflamed bowel disease disappeared due to Nrf2 activation, which was measured by high genetic expression of Nrf2-related proteins such as GCL, GSTS, and SOD.

### 5.10. Vitis vinifera

Wang et al. [26] worked with a DSS-induced C57BL/6 mice model of colitis to evaluate the effects of a seed proanthocyanidin extract from *Vitis vinifera*. Before treatment, the animals presented diminished body weight, elevated diarrhea and bloody stool, and elevated levels of pro-inflammatory cytokines (IL-1β, IL-6, TNF-α), pro-oxidative enzymes, and NF-κB-related proteins expression, as well as decreased levels of HO-1 and SOD. After treatment, the mice started to present elevated HO-1 levels, elevated Nrf2-related protein gene expression, and decreased NF-κB-related protein expression.

### 5.11. Acanthopanax senticosus

Su et al., 2022 [27] evaluated in vivo and in vitro the effects of a flavonoid extract from *Acanthopanax senticosus* against intestinal inflammation and colitis. The authors used hydrogen peroxide (H_2_O_2_)-induced RAW 264.7 cells and DSS-induced C57BL/6 mice to show that the activation of Nrf2 by the plant’s extract had a role in alleviating the disease. Before treatment, the models presented elevated inflammatory (elevated Keap1 levels), oxidative stimuli (elevated MDA and decreased catalase (CAT), SOD, GPx, and HO-1 levels), and elevated DAI scores in vivo. After treatment, the models presented decreased Keap1 and elevated HO-1 expression, which involves augmented nuclear translocation of the Nrf2.

### 5.12. Forsythia suspensa

Chao et al. [28] studied the effects of a polyphenol-rich *Forsythia suspensa* extract, a well-known traditional medicine in Asian countries such as China, Japan, and Korea, against colitis induced by DSS in C57BL/6 mice. Before treatment with the extract, the mice presented elevated DAI, intestinal villi degeneration, intestinal necrosis, elevated proliferation and infiltration of inflammatory cells in the intestine, and diminished body weight. Additionally, the mice presented augmented levels of IL-1β, MDA, and MPO, as well as reduced levels of SOD. After treatment with the extract, the situation was reversed, and the mice started to present augmented Nrf2 activity measured by decreased levels of MDA, MPO, caspase-1, IL-1β, Gasdermin D, Keap1 activity and NLR family pyrin domain containing 3 (NLRP3) expression, ROS levels, pyroptosis, and ASC and by increased levels of SOD, HO-1, and NQO1.

### 5.13. Artemisia argyi

Shin et al. [29] used *Artemisia argyi* ethanolic extract, also known as wormwood and Chinese mugwort, to evaluate its effects against the DSS-induced C57BL/6 mice model of colitis. The animals presented elevated DAI, colonic dysplasia, colon barrier disruption, and decreased body weight before treatment. Additionally, the mice presented elevated aspartate aminotransferase (AST), alanine aminotransferase (ALT), IL-6, IL-1β, TNF-α, and COX-2 levels. After treatment, the mice regulated Nrf2. Additionally, the animals presented decreased expression levels of IL-6, IL-1β, TNF-α, p-IκBα, p-NF-kB, COX-2 expression, intercellular adhesion molecule 1 (ICAM-1) expression, MCP1 expression, and iNOS expression, as well as elevated HO-1 levels.

### 5.14. Dendrobium fimbriatum

Wang et al. [30] conducted a study with a DSS-induced mice model of colitis to evaluate the effects of *Dendrobium fimbriatum* polysaccharide extract against this IBD sample. Before treatment, the mice presented elevated DAI and colon barrier disruption, decreased body weight, and augmented NF-κB signaling and Th17/regulatory T homeostasis disruption. After treatment, the mice presented diminished NF-κB signaling activation and Th17/regulatory T homeostasis disruption, as well as increased Nrf2 signaling *activation*.

### 5.15. Pisum sativum L.

Guo et al. [31] studied the effects of *Pisum sativum* L. hull polyphenol extracts in a DSS-induced C57BL/6 mice model of colitis. Before treatment, the animals presented high values of DAI and a high prevalence of diarrhea, blood in the stool, and reductions in colon length and body weight. The mice also presented augmented MPO, diminished claudin-1, occluding, and tight junction protein-1 (ZO-1), increased MDA, TNF-α, IL-1β, and IL-6 expressions, and decreased SOD, CAT, total antioxidant capacity (T-AOC), and IL-10 levels, as well as increased Keap1 and decreased Nrf2, GCLC, HO-1, and NQO1 expression levels. After treatment, the animals presented elevated Nrf2 expression and nuclear translocation, GCLC, HO-1, NQO1, IL-10, SOD, CAT, T-AOC, claudin-1, occludin, and ZO-1 expression, in addition to decreased MPO, MDA, TNF-α, IL-1β, IL-6, and Keap1 expressions.

### 5.16. Tetrastigma hemsleyanum

Wu et al. [32] studied the effects of *Tetrastigma hemsleyanum* leaf bio compounds and metabolites in a DSS-induced mice model of colitis. Before treatment, the animals presented diminished colon length and body weight, as well as reduced claudin-1, occludin, ZO-1, IL-10, Nrf2 nuclear translocation, SOD, CAT, HO-1, NQO1, and GCLC levels, in addition to augmented MPO, MDA, IL-1β, IL-6 and TNF-α levels. After treatment, the animals showed elevated Nrf2 nuclear translocation, increased claudin-1, occluding, ZO-1 expressions, IL-10, SOD, CAT, HO-1, NQO1, and GCLC expressions. The mice also presented reduced IL-1β, IL-6, TNF-α, and MPO expression.

### 5.17. Rhus chinensis Mill

Zhang et al. [33] conducted a study with a DSS-induced mice model of colitis to evaluate the effects of *Rhus chinensis* Mill. fruit ethanolic extract. Before colitis induction, the mice presented elevated DAI, colon barrier disruption, and diminished colon length. Additionally, the animals presented elevated MPO, MDA, IL-1β, IL-6, TNF-α, p-NF-kB, p-IκB, COX-2, iNOS, p-P38, p-ERK1/2, and p-JNK levels, as well as reduced SOD, GSH, claudin-1, occludin, ZO-1, Nrf2 activation, NQO1, and HO-1. After treatment, the mice presented reduced MPO, MDA, IL-1β, IL-6, TNF-α, p-NF-kB, p-IκB, COX-2, iNOS, p-P38, p-Erk1/2, and p-JNK expression, as well as elevated SOD, GSH, claudin-1, occludin and ZO-1 expression, Nrf2 activation, and NQO1 and HO-1 expression.

### 5.18. Crocus sativus

Singh et al. [34] conducted a study with a DSS-induced C57BL/6 mice model of colitis to evaluate the effects of *Crocus sativus* aqueous extract against this model of IBD. The animals presented elevated DAI, diminished body weight and colon length, augmented TNF-α and IFN-γ levels, and decreased HO-1, GPX-2, and Nrf2 expression and activity. After treatment, the mice presented elevated Nrf2 expression and activity, increased HO-1 and GPX-2, and decreased TNF-α and IFN-γ expression levels.

### 5.19. Prunus mahaleb

Ferramosca et al. [35] studied a DSS-induced BALB/c mice model of colitis to evaluate the effects of *Prunus mahaleb* concentrated fruit extract. Although the mice presented elevated intestinal inflammation and OS marked by decreased Nrf2 expression and activity, as well as diminished GSH, GSR, and glucose-6-phosphate dehydrogenase (G6PD) before treatment, the use of the fruit extract reverted this situation and augmented the Nrf2 expression and activity of the treated animals, as well as their expression of GSH, GSR, and G6PD.

### 5.20. Quercus ilex L.

Castejón et al. [36] studied a trinitrobenzene sulfonic acid (TNBS)-induced Wistar mice model of colitis to evaluate the effects of *Quercus ilex* L. leaf polyphenolic extract in the activation of the Nrf2 signaling pathway. Before treatment, the mice presented elevated anorexia, intestinal adhesions to adjacent organs, weight/length ratio of the colon, transmural necrosis, edema, diffuse inflammatory cells infiltration, ulceration, and crypt distortion, as well as diminished body weight. Additionally, the mice presented increased MPO, TNF-α, IL-1β, COX-2, iNOS, IκB-α degradation, p65 nuclear translocation, p50 nuclear translocation, NF-κB-mediated transcriptional protein activation, and MAPK (JNK, p38, and ERK1/2) protein activation, in addition to decreased HO-1 and Nrf2-mediated transcriptional protein activation. After treatment with the plant and further increased Nrf2-mediated transcriptional protein activation and HO-1 overexpression, the mice presented decreased MPO, TNF-α, IL-1β, COX-2, and iNOS expression, IκB-α degradation, p65 and p50 nuclear translocation, NF-κB-mediated transcriptional protein activation, and MAPK (JNK, p38, and ERK1/2) protein activation.

### 5.21. Ziziphus spina-christi

Almeer et al. [37] studied an AcOH-induced Wistar mice model of colitis to evaluate the effects of *Ziziphus spina-christi* fruit extract in activating Nrf2 signaling. Before treatment, the mice presented elevated weight/length ratio of the colon, necrosis, ulceration, corrosion, hemorrhagic diarrhea, intestinal focal infiltration of inflammatory cells, crypt distortion, and decreased intestinal mucin concentration. The mice also presented elevated MPO, lipid peroxidation (LPO), NO, GSH, TNF-α, IL-1β, iNOS, COX-2, and NF-κB activation and signaling, in addition to decreased SOD, CAT, GSR, GPx, and Nrf2/HO-1 activation and signaling levels. After treatment with the fruit extract, there was diminished p38, MAPK, and VEGF-A expression, Bax and caspase-3-mediated apoptosis, MPO, LPO, NO, TNF-α, IL-1β, iNOS, and COX-2 expression, as well as increased GSH, SOD/, CAT, GSR, GPx and Nrf2/HO-1 activation and signaling. The treated mice also presented diminished NF-κB activation and signaling.

### 5.22. Perilla frutescens

Park et al., 2017 [38] studied a DSS-induced ICR mice model of colitis to evaluate the effects of *Perilla frutescens* extracts against this model of IBD. The animals presented diminished body weight and colon length, increased DAI, diarrhea, bloody stool, and intestinal infiltration of inflammatory cells. Before treatment, the animals also presented elevated COX-2, iNOS, cyclin D1, NF-κB activation and translocation, IκBα phosphorylation and degradation, p65 nuclear translocation, STAT3 signaling, TLR4, Interferon regulatory factor 3 (IRF3) (an inducer of interferon production), IFN-α, and IFN-γ, in addition to reduced levels of Nrf2/HO-1 activation and signaling and of HO-1. After treatment, the animals showed elevated levels of Nrf2/HO-1 activation and signaling and HO-1 expression, as well as decreased COX-2, iNOS, and cyclin D1 expressions, NF-κB activation and translocation, IκBα phosphorylation and degradation, p65 nuclear translocation, STAT3 signaling and TLR4, IRF3, IFN-α, and IFN-γ expression.

## 6. Phytochemicals Targeting Nrf2 Pathways

### 6.1. Bioactive 6-Shogaol

Zhang et al., 2018 [39] conducted both an in vitro and in vivo study to evaluate the effects of 6-shogaol, a bioactive constituent of *Zingiber officinale* Roscoe, on colitis-associated Raw 264.7 macrophage cells, colon-26 LPS-activated cells, and a DSS-induced FVB/NJ mice model of colitis. Before treatment, the models presented elevated inflammation and OS measured by elevated levels of pro-inflammatory cytokines (TNF-α, IL-6, IL-1β) and lipocalin (Lcn) 2. After treatment, the animals had activated Nrf2 signaling; therefore, the associated diseases were ameliorated by increased HO-1 levels and decreased TNF-α mRNA, IL-6 mRNA, IL-1β mRNA, and iNOS mRNA levels.

### 6.2. Licochalcone A

Liu et al. [40], during an in vivo study using a DSS-induced C57BL/6 mice model of colitis, demonstrated the effects of licochalcone A, a major compound of *Glycyrrhiza* species, against Nrf2 deactivation during IBD. The mice presented intestinal morphological alterations due to increased inflammatory and oxidative damage. Furthermore, the rats showed elevated MPO, TNF-α, IL-1β, IL-6, COX-2, NF-κB p65, IKKα, p-IκB, NO, and Keap1 expression levels, as well as decreased SOD and GSH levels. After treatment, the mice presented activation of Nrf2 due to increased HO-1 and GCL and decreased Keap1, NF-κB- p65, IKKα, and p-IκB expression levels.

**Figure 2:**
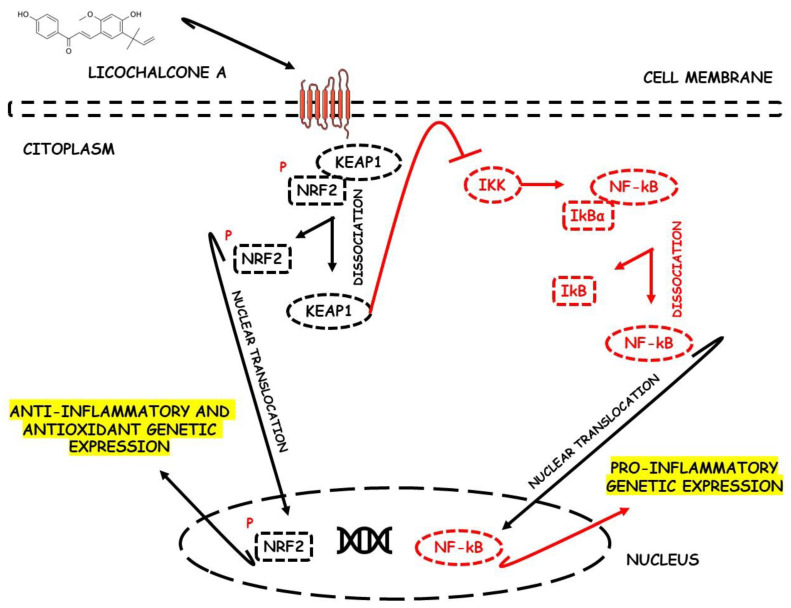
Possible therapeutic targets for chalcones against the IBD-related NF-kB and Nrf2 dysregulation. IkB, inhibitor of nuclear factor-kB; IkBα, nuclear factor of kappa light polypeptide gene enhancer in B-cells inhibitor, alpha; IKK, inhibitor of nuclear factor-κB (IκB) kinase; KEAP1, Kelch-like ECH-associated protein 1; NF-kB, nuclear factor kappa b; Nrf2, nuclear factor erythroid 2–related factor 2.

Figure 2 shows the possible therapeutic targets for chalcones against IBD-related NF-kB and Nrf2 dysregulation.

### 6.3. Oligonol

Kim et al. [41] evaluated the effects of oligonol using a mouse colitis model. Before treatment, the mice showed intestinal mucosal dysfunction and increased OS parameters, notably through increased NF-κB activation and TNF-α, IL-6, IL-1β, COX-2, iNOS, ROS, JNK, and M1 macrophages. After treatment, the mice showed increased activation of Nrf2 through decreased activation of NF-κB-p65 and increased levels of HO-1, aldo-keto reductase (AKR), GSTs, and Nrf2 genetic transcription.

### 6.4. Cyanidin-3-O-Glucoside

Ferrari et al. [42] conducted an in vitro study with Caco-2 human intestinal epithelial cells exposed to TNF-α as a model of intestinal inflammation to evaluate the roles of cyanidin-3-O-glucoside in this model of IBD. Before treatment, the cells showed elevated NF-κB activation measured by TNF-α-induced nuclear translocation of NF-κB-p65 and increased expression of pro-inflammatory cytokines such as IL-6. Furthermore, the rise in COX-2, Prostaglandin E2 (PGE2), thromboxane 2, leukotrienes, ROS, and decreased GSH were also measured. After treatment with cyanidin-3-O-glucoside extract, the cells showed reduced NF-κB-p65 translocation and activation of the Nrf2 pathways measured by increased Nrf2 nuclear translocation and HO-1 and NQO1 expression.

### 6.5. Luteolin

Li et al. [43] conducted a study involving a DSS-induced mice model of colitis to investigate the effects of luteolin. Before treatment, the animals had elevated intestinal inflammation (elevated expression of TNF-α, IL-6, and iNOS), intestinal OS (elevated MDA), prominent mucosal infiltration of inflammatory cells, and activation of NF-κB signaling. After treatment, luteolin promoted an increase in Nrf2 activation and expression. In addition, the compound promoted increased expression of NQO1, HO-1, SOD, and CAT.

### 6.6. Alpinetin

Tan & Zheng [44] conducted a study involving a DSS-induced C57BL/6 mice model of colitis to evaluate the effects of alpinetin. Before treatment, the animals showed increased DAI, intestinal OS, and inflammation (elevated levels of TNF-α, IL-1β, and IL-6) and increased ROS, MPO, and MDA expression. After alpinetin treatment, the mice showed increased Nrf2 activation and signaling and increased levels of HO-1 and SOD.

### 6.7. Cardamonin

Wang et al. [45] evaluated whether cardamonin could significantly improve the disease process in the models of IBD. Before treatment, the animals showed damage to the intestinal mucosa, increased DAI, and elevated intestinal oxidative and inflammation parameters (TNF-α, IL-6, IL-1β, and mitochondrial ROS), notably through increased NF-κB activation and signaling. After treatment, mice showed increased activation of Nrf2 and increased levels of SOD, HO-1, and NQO1, also reverting the previous inflammatory process.

### 6.8. Puerarin

Jeon et al. [46] studied the effects of puerarin in a DSS-induced Balb/c mice model of colitis. The animals were reported to have elevated DAI, intestinal inflammation, and OS (elevated MPO, TNF-α, IL-6, IFN-α, IL1β, ROS, COX-2, PGE2, iNOS, NO, MDA, GSH, and ZO-1) and prominent infiltration of inflammatory cells. After treatment, the use of puerarin increased the activity of the Nrf2 signaling pathway through increases in NQO1, HO-1, SOD, and CAT expressions and reduced the activation of the NF-κB pathways.

### 6.9. Gallic Acid

Pandurangan, Mohebali et al. [47] evaluate the effects of gallic acid in a DSS-induced Balb/c mouse colitis model. The study revealed that mice before treatment had elevated intestinal OS (elevated MDA), increased levels of pro-inflammatory cytokines in the intestine (such as IL-21 AND IL-23), and increased colon size. After treatment, gallic acid increased the expression of Nrf2 and its signaling, and promoted elevated levels of SOD, CAT, NQO1, and UDP-glucuronyl transferase (UDP-GT) and, additionally, reduced the levels of NF-κB activation and signaling.

### 6.10. Sulforaphane

Alattar et al. [48] conducted a study involving a mouse model of DSS-induced colitis to analyze the effects of sulforaphane against this IBD model. Before treatment, the animals showed increased intestinal inflammation, intestinal OS, inflammatory mucosal cell infiltration, and NF-κB activation and signaling. Furthermore, it was possible to observe increased expression of PGC, mitochondrial transcription factor A (TFAM), and mTOR. After treatment, sulforaphane promoted an increase in the expression and signaling of Nrf2 and elevated the expression of HO-1.

### 6.11. Asperuloside

Chen et al. [50] conducted an in vivo and in vitro study to evaluate the role of asperuloside in relieving colitis. The studied mice showed high DAI, increased colon length, increased OS, and increased levels of inflammatory cell mucosa infiltration. Both models expressed increased TNF-α, IL-6, IL-10, ROS, MPO, and MDA levels. After treatment, the models showed decreased p-NF-κB-p65 expression and increased SOD, Nrf2 expression and signaling, NQO1 mRNA, and HO-1 mRNA levels. 

### 6.12. Syringin

Zhang et al. [51] studied a DSS-induced Balb/c mice model of colitis to evaluate the effects of syringin. The animals were reported to have intestinal inflammation (elevated TNFα, IL-6, IL1β, and COX-2), intestinal OS, prominent transmural inflammatory cell infiltration, and increased iNOS levels. In addition, the authors observed a decrease in ZO-1 and occludin protein expression. After treatment with syringin, there was a decrease in p-NF-κB, IκBα, and p-IκBα expression and an increase in Nrf2 activation and signaling, as well as elevated HO-1 expression.

### 6.13. Paeoniflorin

Wu et al. [52] worked on an in vitro study with LPS-induced Caco-2 intestinal epithelial cells (colitis model) to evaluate the effects of paeoniflorin against Nrf2 deactivation during this model of IBD. Before treatment, Caco-2 cells showed a high inflammatory process, evidenced by an increase in TNF-α, IL-6, COX-2, and iNOS, in addition to a decrease in ZO-1. With the use of paeoniflorin, the results showed an increase in Nrf2 activation and signaling expression, culminating in elevated HO-1 and decreased p-NF-κB expression.

### 6.14. Dehydrocostus Lactone

Yuan et al. [53] conducted an in vivo and in vitro study to assess the role of dehydrocostus lactone in colitis relief. The studied mice showed high DAI. Both models expressed increases in TNF-α, IL-1β, iNOS, ROS, and COX-2 levels and decreased ZO-1 expression. After treatment, the models showed a decrease in the expression of p-NF-κB p-65, Keap1, and MAPK and an increase in the expression of IKKα/β, Nrf2, and HO-1.

### 6.15. Leonurine

Qi et al. [54] studied a DSS-induced C57BL/6 mouse model of colitis to analyze the effects of leonurine against this IBD model. The animals showed increased DAI, high inflammation, and pro-inflammatory cytokines (such as TNF-α, IL-6, and IL-1β) and a reduction in GSH before treatment. After treatment, there was an increased activation and signaling of the Nrf2 pathways, increased expression levels of HO-1 and SOD, and decreased expression of p-NF-κB, TLR4, and pro-inflammatory cytokines.

### 6.16. Crocin

Khodir et al. [55] considered an in vivo study that evaluated the antiulcerogenic and coloprotective properties of crocin, the main biologically active agent of saffron, against an acetic acid (AA)-induced mice model of colitis. Due to the use of AA, the rat showed an increase in DAI, stromal edema, ulcerated mucosa, increased OS, and infiltration of inflammatory cells in the intestine. There was a reduction in serum total antioxidant capacity (TAC), CAT, SOD, GSH, HO-1, and Nrf2, in addition to an increase in TNF-α and many inflammatory cytokines. After using crocin, there was a reduction in oxidative, inflammatory, and apoptotic activity and an increase in antioxidant defenses. There was also a reduction in TNF-α, caspase-3, MDA, and ROS and an increase in Nrf2, HO-1, TAC, CAT, SOD, and GSH, and improvements in the animals clinically.

### 6.17. Quercetin

Khater et al. [56] performed an in vivo study to verify the antioxidant and anti-inflammatory effects of quercetin, the main polyphenolic flavonoid in various fruits and vegetables, in relieving a DSS-induced Balb/c and Kunming mice model of colitis. Before DSS administration, rats showed increased DAI, inflammatory cell infiltration, crypt loss, mucin and goblet cell depletion, and ulceration with coagulative epithelium necrosis. Furthermore, there was an increase in the levels of ROS, MPO, IL-6, TNF-a, IFN-γ, COX-2, MDA, NF-κB, inflammatory cytokines, and proteolytic enzymes, with a reduction of TAC, GSH, SOD, and CAT, and lower levels of tight junction protein genes. After therapy with quercetin, there was a reduction in DAI, improvement in antioxidant function, increased genetic expression of Nrf2 and HO-1, upregulation of genes encoding occludin, Mucin 2-oligomeric mucus gel-forming (MUC2), and junctional adhesion molecule (JAM), and inhibition of transcription factors that activate NF-κB.

### 6.18. OPAL, 8-Oxypalmatine

Cheng et al. [57], during an in vivo study, comparatively investigated the potential effect and mechanism of 8-oxypalmatine (OPAL) and palmatine (PAL) in a DSS-induced Balb/c mice model of colitis. OPAL is a metabolite derived from PAL, an alkaloid from *Fibraureae caulis* Pierre. During the study, after using DSS, there was an increase in DAI, weight loss, massive infiltration of inflammatory cells, destruction of crypts, thickening of the intestinal mucosa, and an increase in inflammatory mediators in the colon. There was an activation of NLRP3, an increase in TNF-a, IL-1B, IFN-γ, IL-17A, IL-6, MDA, MPO, ASC, caspase-1 and NO, and a reduction in IL-10 levels, TAC, SOD, GSH, CAT, GSH-Px, NRF2, and HO-1. The use of OPAL provided greater expression of mRNA and proteins of the Nrf2 and HO-1 pathways, in addition to inhibiting the activation of the NLPR3 inflammasome and the expression of mRNA of the NLRP3 proteins, ASC, and caspase-1. With the use of OPAL, there was also an attenuation of clinical manifestations, reduction in DAI, and less pathological damage and markers of OS and intestinal inflammation.

### 6.19. PMID, 3-(3-Pyridylmethylidene)-2-Indolinone

Wang et al. [58] performed an in vivo study to evaluate the anticolitis effects of pre-treatment with 3-(3-pyridylmethylidene)-2-indolinone (PMID), which is a synthesized derivative of 2-indolinone compounds, in DSS-induced C57BL/6J and ICR mice models of colitis. In mice, colitis caused greater DAI, reduced colon length, inflammatory cell infiltration, crypt loss, and epithelial cell necrosis. In addition, there were higher levels of IL-6, TNF-α, MCP-1, IFN-γ, and p65, and lower expression levels of genes related to the Nrf2 pathways. Pre-treatment with PMID ameliorated weight loss, DAI, and inflammation, inhibited NF-κB activation, diminished TNF-α, IFN-γ, IL-6, and p65 levels, caused activation of the Nrf2/ARE pathways, and augmented the expression of NQO1 and HO-1, among other Nrf2-related enzymes.

### 6.20. Ruscogenins

Using an in vivo and in vitro study, Wen et al. [59] evaluated the therapeutic effects of ruscogenins (RUS), a steroidal sapogenin derived from the Chinese herb *Radix Ophiopogon japonicus* widely used for treating inflammation and cardiovascular diseases, in the activation of Nrf2 to improve the function of the intestinal barrier. During the in vivo study with a DSS-induced C57BL/6J mice model of colitis, before the DSS administration, there was an increase in DAI, intestinal barrier dysfunction, abscesses, and crypt ulcers, higher levels of intestinal inflammation, apoptosis, and bacterial translocation and increased levels of TNF-a, IFN-y, NLRP3, caspase-1 and 11, GSDMD, Bax, and caspase-3. After using RUS, the mice had improved damage induced by DSS and more remarkable survival, in addition to activation of the Nrf2/NQO1 pathway, higher levels of B-cell lymphoma 2 (Bcl-2) protein, and attenuation of the Bax/c-caspase-3 pathway. During the in vitro study, the inflammatory injury was caused by LPSs in rat colon organoids. The organoids presented increased TNF-α and IFN-γ, factors related to macrophage migration, higher expression of Bax and caspase-3, increased apoptosis, and reduced Bcl-2 expression. On the other hand, with the use of RUS, there was an increase in the expression of Nrf2 and NQO1 proteins, as well as a reduction in the expression of TNF-α and IFN-γ and of factors related to macrophage migration.

### 6.21. Caffeic Acid

Wan et al. [60], during an in vivo study with a mice model of colitis, evaluated the possible protective effects of caffeic acid (CA), one of the main phenolic acids in coffee, against the DSS-induced mice model of colitis. The administration of DSS generated an increased DAI and greater intestinal permeability with inflammatory cell infiltration and disruption of the intestinal barrier, stimulation of OS, weight loss, colon shortening, intestinal dysbiosis, and a reduction in the number of goblet cells. Such modifications were followed by an increase in IL-6, TNF-α, IL-1β, IL-12, MDA, ROS, and other inflammatory cytokines, and reduced levels of IL-10, GSH-Px, SOD, CAT, ZO-1, and occludin. During the experiment, the use of CA provided greater mRNA expression of Nrf2-related proteins, HO-1, NQO1, occluding, and IL-10, higher transcriptional levels of SOD, GPX1, GPX2, and CAT, reduced mRNA expression of IL-1β, IL-6, and TNF-α, and lower MDA levels.

### 6.22. Schisandrin B

W. Zhang et al. [61], during an in vivo and in vitro study, evaluated the role and molecular mechanisms of relieving colitis using schiandrin B, the main ingredient of *Schisandra chinensis*. The in vivo study used a DSS-induced mice model of colitis which demonstrated increased DAI, formation of intestinal ulcers, dysfunction of intestinal integrity and permeability, greater bacterial translocation, inflammatory infiltration, and increased levels of TNF-α, IL-6, IL-18, and IL-1β before treatment. After using schisandrin B, there was a reduction in the inflammasome NLRP3 activation and in the production of TNF-α, IL-6, IL-18, IL-1β, caspase, and GSDMD with simultaneous activation of the AMPK/NRF2 pathway and reduction of ROS-induced mitochondrial damage. During the in vitro study, there was stimulation of inflammatory injury in human colon cancer cell line (HCT)-116 cells by LPSs, which caused an increase in the levels of TNF-α, IL-6, IL-18, IL-1β, NLRP3 activation, ROS, GSDMD, apoptosis, and mitochondrial damage, as well as increased lactate dehydrogenase (LDH) activity. With the use of schisandrin B, there was a reduction in all inflammatory cytokines expression that had previously been increased, in addition to greater expression of protein p-AMPK and Nrf2, with a reduction in oxidative mitochondrial damage and a relative improvement in colitis.

### 6.23. GB1a

Yu et al. [62] performed an in vivo study to explore the therapeutic effects of GB1a, the main active component of *Garcinia kola* nuts, against a mice model of colitis. The induction of colitis in rats with DSS caused weight loss, increased DAI, a reduced number of crypts, increased inflammatory infiltration, and increased synthesis of ROS and pro-inflammatory cytokines. After the administration of GB1a, there was a reduction in the NF-κBp65 translocation to the nucleus with inhibition of the NF-κB pathway, a decrease in the levels of MPO, MDA, IL-6, and TNF-α mRNA, and diminished levels of chemokines such as CCL5, CCL20, and CXCL1. Concomitantly, there was an increase in the translocation of Nrf2 to the nucleus and its expression, higher production of HO-1, GSH, and SOD, and higher levels of ZO-1 mRNA and occludin mRNA.

### 6.24. Diosmetin

Li et al. [63], during in vivo and in vitro studies, aimed to verify the effects and potential molecular mechanisms of diosmetin in the treatment of IBD, as it is a natural flavonoid compound of citrus species. The use of DSS to induce colitis in rats caused increased DAI, weight loss, colon shortening, inflammatory infiltration, OS, increased permeability, and intestinal epithelial barrier dysfunction. The administration of diosmetin inhibited oxidative damage, with a reduction in the levels of MDA, NF-κB activation, and expression of the inflammatory cytokines, in addition to increasing the levels of Nrf2 activation, HO-1, Sirt-1, GSH-Px, GSH, SOD, and ZO-1. In vitro, Caco-2 and IEC-6 cells presented inflammatory lesions stimulated by LPSs, with increased ROS levels and reduced claudin and occludin. After administration of diosmetin, there was an increase in the expression of claudin-1, occludin, ZO-1, Nrf2 activity, and HO-1, along with a decline in ROS and NF-κB activation levels. 

### 6.25. Atractylenolide III

Ren et al. [64] carried out an in vivo study to investigate the therapeutic effects of various actives of *Atractylodes macrocephala*, including Atractylenolide III, in the TNBS-induced mice model of colitis. With TNBS, the animals presented increased DAI, intestinal inflammation, weight loss, colon shortening, infiltration of inflammatory cells, OS, submucosal necrosis, distortion of the mucosal structure, and ulcers, along with increased MPO, IL-1β, TNF-α, MDA, Formyl peptide receptor 1 (FPR1), NADPH (nicotinamide adenine dinucleotide phosphate hydrogen) oxidase 1 (NOX1), and phosphorylated Nrf2, as well as reduced levels of Nrf2 activation and signaling, SOD, GSH-Px, and CAT. After treatment with atractylenolide III, there was a reduction in the MPO, FPR1, NOX1, phosphorylated Nrf2, MDA and IL-1β, and TNF-α mRNA expressions, as well as increases in FPR1, Nrf2 activation, and signaling, CAT, SOD, and GSH-Px.

### 6.26. Polydatin

G. Chen et al. [65] worked with a DSS-induced C57BL/6 mice model of colitis and LPS-treated RAW264.7 cells to evaluate the effects of *Polydatin*, a bioactive compound isolated from the root and rhizome of *Polygonum cuspidatum*. Before treatment, the animals presented elevated DAI and OS, increased secretion of pro-inflammatory cytokines, and reduced colon length. After treatment, there was elevated nuclear translocation and phosphorylation of Nrf2, diminished phosphorylation of Erk1/2 and JNK1/2, and increased expression of HO-1, NQO1, and IL-10, as well as increased Akt phosphorylation. Peritore et al. [66] also studied the same bioactive constituents of *Polygonum cuspidatum* but evaluated its roles against IBD in a DNBS-induced CD1 mice model of colitis. In this study, the mice presented, in addition to intestinal inflammation and OS, prominent transmural inflammatory cell infiltration and ulcer formation. Reduced NF-κB translocation, IkBα degradation, and elevated levels of SIRT1, Nrf2, and HO-1 expressions were highlighted with the treatment.

### 6.27. Rosmarinic Acid

Marinho et al. [67] conducted a study involving a DSS-induced C57BL/6 mice model of colitis to analyze the actions of a polyphenol called *Rosmarinic Acid* against this model of IBD. The mice presented elevated DAI, intestinal inflammation, prominent transmural inflammatory cell infiltration, and elevated expression of inflammasome-related proteins. After treatment, the animals expressed elevated Nrf2 and HO-1 and reduced pro-inflammatory and OS genetic expression.

### 6.28. Imperatorin

Luo & Luo [68] worked with a TNBS-induced SD mice model of colitis to evaluate the effects of *Imperatorin*, an active furocoumarin of *Angelica dahurica*, against this model of IBD. Before treatment, the animals presented elevated DAI, intestinal inflammation, and OS, coursing with increased TNF-α and IL-6 expression levels. The rats expressed elevated Nrf2 levels, increased ARE and HO-1, and reduced OS genetic expression with treatment.

### 6.29. Berberine

Jia et al. [69] conducted a study involving an AcOH-induced Wistar mice model of colitis to analyze a natural isoquinoline alkaloid known as berberine. The rats presented intestinal inflammation, prominent transmural inflammatory cell infiltration, mucosal edema, increased MPO and PGE2, and elevated tissue necrosis. After treatment, the animals presented elevated mRNA expression of p38MAPK, elevated Nrf2/HO-1 signaling pathway, and decreased OS-related gene expression and apoptotic activity.

### 6.30. Curcumin

Yin et al. [70] worked with hypoxia and hypothermia-induced NEC rat model of colitis to evaluate the anti-colitis effects of curcumin, a diketone component extracted from the rhizomes of *Zingiberaceae* and *Araceae* plants. Before treatment, the mice presented elevated intestinal inflammation and secretion of pro-inflammatory cytokines. After the use of curcumin, the authors reported an increase in the SIRT1/Nrf2 pathway, a reduction in TLR4 expression, and also a decrease in pro-inflammatory genetic expression.

### 6.31. Sesamin

Bai et al. [71] conducted a study involving Caco-2 human intestinal epithelial cells exposed to H2O2 to evaluate the actions of sesamin, a lignan derived from sesame seeds. Before treatment, elevated intestinal inflammation was observed (increased IL-1β, IL-6, TNF-α, and M1 macrophages) along with increased OS, and increased intestinal cytotoxicity. After treatment with *Sesamin*, there was a decrease in Keap1 levels, and it promoted an increase of the Nrf-2/ARE expression and induced the expression of p-AKT/AKT, GCLC, glutamate-cysteine ligase modifier subunit (GCLM), NQO1, and HO-1.

### 6.32. Toosendanin

Fan et al. [72] worked with toosendanin, a triterpenoid extracted from the bark of the fruit of *Melia toosendan*. This study used a DSS-induced C57BL/6 mice model of colitis. Before treatment, the mice presented elevated DAI, increased intestinal inflammation, and increased OS (elevated ROS). After using the compound, the animals presented decreased pro-inflammatory and OS gene expression as soon as toosendanin promoted an increase in Nrf2 and HO-1 expression levels.

### 6.33. Galangin

Sangaraju et al. [73] studied LPS-treated RAW264.7 cells and a DSS-induced Balb/c mice model of colitis to analyze the actions of a phenolic phytochemical isolated from *Propolis* and *Alpinia ofcinarum*, known as galangin, against these models of IBD. The animals presented elevated DAI, the loss of epithelial and goblet cells, crypt aberrations, increased levels of pro-inflammatory cytokines (IL-6 and TNF-α), increased NF-κB activation, and elevated OS. After treatment, galangin induced an increase in the Nrf2 signaling pathway activation, as well as increases in HO-1, decreases in pro-inflammatory genetic expression, reductions in JNK phosphorylation, and diminished p-IKKα/β expression levels.

### 6.34. Apocynin

Hwang et al. [74] conducted a study involving a DSS-induced Balb/c mice model of colitis to evaluate the effects of apocynin, a phytochemical extracted from the root of the medicinal herb *Picrorhiza kurroa.* The animals presented increased intestinal OS and intestinal inflammation, as well as elevated COX-2 expression. After using apocynin, the mice presented an increase in the Nrf2 signaling pathway activation, upper levels of HO-1, and decreased pro-inflammatory genetic expression.

### 6.35. Hesperidin

Guo et al. [75] worked with a DSS-induced C57BL/6 mice model of colitis and TNF-α/IFN-γ-treated Caco-2 cells to evaluate the effects of hesperidin. The models presented elevated inflammation and an increase in pro-inflammatory cytokines, reduced levels of IL-10, and elevated levels of MPO. After treatment with hesperidin, there was an increase in the Nrf2 antioxidant signaling pathway activation, upper levels of NQO1, HO-1, and regulatory T cells, decreased levels of MPO, diminished pro-inflammatory cytokines expression, and increased IL-10 expression in the colonic tissues.

### 6.36. Norisoboldine

Lv et al. [76] conducted a study involving a TNBS-induced Balb/c mice model of colitis to evaluate the effects of norisoboldine, the main isoquinoline alkaloid ingredient of *Radix linderae*. The animals presented elevated intestinal inflammation marked by increased levels of pro-inflammatory cytokines and an increased OS. After treatment, the rats expressed Nrf2 signaling pathway activation and decreased NLRP3 inflammasome activation.

### 6.37. Hyperoside

L. Yang et al. [77] worked with a DSS-induced C57BL/6 mice model of colitis to analyze the effects of a flavonoid glycoside compound known as hyperoside against this model of IBD. Before treatment, the animals presented elevated DAI, intestinal inflammation, intestinal OS, and a reduction in the colon length. After treatment, the Nrf2 signaling pathway activity increased, with elevated levels of HO-1 and SOD mRNA expression and decreased levels of NF-κB.

### 6.38. Glyceollins

Seo et al. [78] studied a DSS-induced Balb/c rat model of colitis to evaluate the effects of glyceollins, which are derived from daidzein in soybean. It was reported that the animals presented elevated DAI, intestinal inflammation, intestinal OS, prominent transmural inflammatory cell infiltration, and increased mucosal necrosis. After treatment, glyceollins increased the Nrf2 signaling pathway activity, reduced NF-κB expression, and decreased other pro-inflammatory gene expressions.

### 6.39. Carnosic Acid

Yang et al. [79] conducted a study involving a DSS-induced mice model of colitis to analyze the effects of carnosic acid, a naturally polyphenolic diterpene derived from the rosemary plant. Before treatment, the animals presented elevated DAI, intestinal inflammation (elevated expression of TNF-α, IL-17A, IL-6, IFN-γ, IL-1β, and IL-18), intestinal OS, prominent transmural inflammatory cells infiltration, and NF-κB activation. After treatment, there was increased Nrf2 expression, a reduction of Nrf2 ubiquitination, and further degradation. Additionally, the bioactive compound promoted a reduction in the levels of the NLRP3 inflammasome, increased GCLM, NQO1, and HO-1 expression, and promoted a reduction in OS gene expression among the intestine cells, as well as decreased Keap1 levels.

### 6.40. Protocatechuic Acid

Crespo et al. [80] worked with a TNBS-induced Balb/c mice model of colitis to evaluate the effects of protocatechuic acid. This phenolic acid can be found in many edible vegetables, fruit, and nuts. The study revealed that the mice presented elevated intestinal inflammation, intestinal OS, increased pro-inflammatory cytokines (such as IL-6, TNF-α, and IL-1β), and elevated COX-2 expression before treatment. After treatment, protocatechuic acid increased Nrf2 expression, reduced COX-2 expression, and decreased NF-κB levels.

## 7. Medicinal Plants and Phytochemicals Targeting the Nrf2 Signaling Pathway during IBD-Related Colorectal Cancer Models

**Figure 3:**
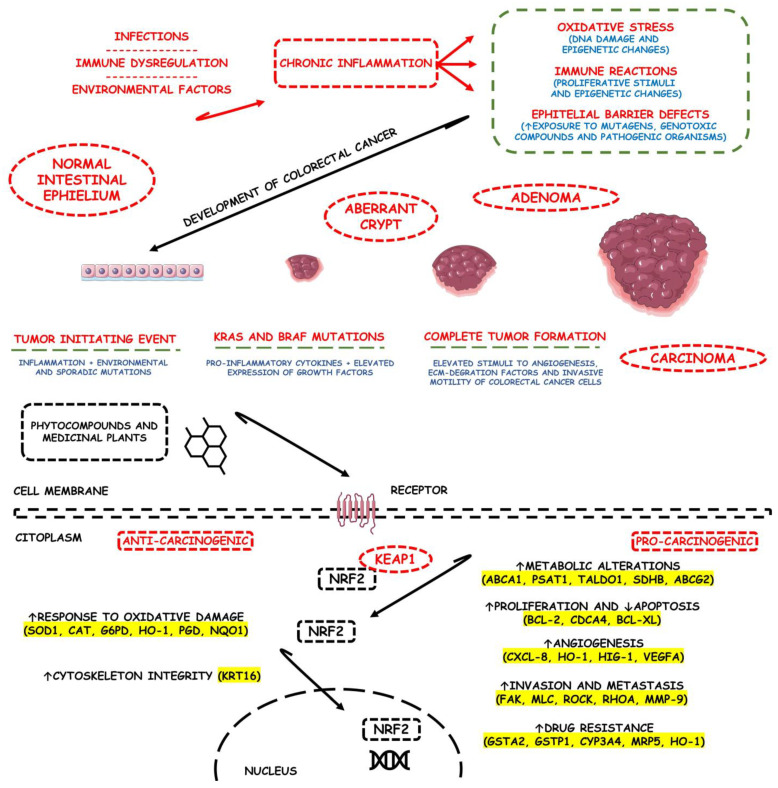
Physiopathology of colitis-associated tumorigenesis and the roles of Nrf2 in this condition. ↑, increase; ↓, decrease; ABCA1, ATP-binding cassette transporter ABCA1; ABCG2, ATP Binding Cassette Subfamily G Member 2 (Junior Blood Group); BCL-2, B-cell leukemia/lymphoma 2 protein; BCL-XL, B-cell lymphoma-extra-large; BRAF, proto-oncogene B-Raf and v-Raf murine sarcoma viral oncogene homolog B; CAT, catalase; CDCA4, E2F family of transcription factors; CXCL8, interleukin 8; CYP3A4, cytochrome P450 3A4; DNA, deoxyribonucleic acid; ECM, extracellular matrix; FAK, focal adhesion kinase; G6PD, glucose-6-phosphate dehydrogenase; GSTA2, glutathione S-transferase alpha 2; GSTP1, glutathione S-transferase P 1; HIG-1, HIG1 domain family member 1A; HO-1, heme oxygenase 1; KEAP1, Kelch-like ECH Associated Protein 1; KRAS, Kirsten rat sarcoma virus; KRT16, Keratin, type I cytoskeletal 16; MLC, nonmuscle myosin regulatory light chain gene; MMP-9, matrix metalloproteinase 9; MRP5, multidrug resistance protein 5; NQO1, NAD(P)H quinone dehydrogenase 1; Nrf2, nuclear factor erythroid 2–related factor 2; PGD, preimplantation genetic diagnosis; PSAT1, class-V pyridoxal-phosphate-dependent aminotransferase family; RHOA, Ras homolog family member A; ROCK, Rho-associated protein kinases; SDHB, succinate dehydrogenase (ubiquinone) iron-sulfur subunit, mitochondrial; SOD1, superoxide dismutase 1; TALDO1, Transaldolase 1; VEGFA, vascular endothelial growth factor A.

Figure 3 briefly shows the physiopathology of colitis-associated colorectal cancer and suggests the roles of Nrf2 in this condition.

### 7.1. Procyanidin B2

In a mice model of IBD, Zhu et al. [167] evaluated the anticancer effects of procyanidin B2 (PB2) (a member of oligomeric anthocyanins precursors) in the intestinal injury repair and colitis-associated tumorigenesis inhibition. The authors found that PB2 could effectively protect the gut from ROS accumulation and against neoplastic damage from irradiation. These effects were attributed to the PB2 activities on reducing Nrf2 degradation and increase in Nrf2 translocation into the cells’ nucleus. The activation of Nrf2 signaling was also important to promote the Lgr5-positive intestinal stem cells (Lgr5+ISCs) driven regeneration against colorectal carcinogenesis associated with IBD. These cells’ activation was mediated via enhanced Wingless-related integration site (Wnt) / β-catenin signaling, which depends strictly on the Nrf2 signal. The experimental results also highlighted the potent anti-colitis effects of PB2 through suppressing nuclear localization of p65.

### 7.2. Resveratrol

In a DSS-induced mice model of colitis, Zheng et al. [168] studied the anticancer effects of resveratrol in colitis-associated tumorigenesis. The authors highlighted that the activation of the Nrf2-signaling against colorectal cancer development through resveratrol depends on the crosstalk between Nrf2 and the mitogen-activated protein kinase phosphatase 1 (Mpk-1), protecting the animals mainly against intestinal inflammation. In this model of colitis-associated tumorigenesis, the Mkp-1−/− mice exhibited a phenotype similar to Nrf2−/− mice with significantly more tumors. Additionally, the tumors derived from Mkp-1−/− were highlighted with exacerbated macrophage infiltration levels, other inflammatory and immune cells, and increased expression of nitrotyrosine, p53BP1, and markers of OS and DNA damage.

### 7.3. Digitoflavone (Dietary)

Yang et al. [169] studied the anticancer effects of dietary digitoflavone against the Caco-2 cell line in vitro model of H2O2-induced OS colitis-associated colorectal tumorigenesis. The authors suggested that the use of the bioactive compound significantly increased Nrf2 expression, as well as its nuclear translocation and related expression of downstream phase II antioxidant enzymes, which resulted in significant H_2_O_2_-induced OS and cell death via p38 MAPK-Nrf2/ARE pathway inhibition. These results suggested digitoflavone can be considered a protective factor against colorectal carcinogenesis.

### 7.4. Theobroma cacao

Pandurangan, Saadatdoust, et al. [170] studied the antioxidant properties of cocoa against the DSS-induced mice model of colitis-associated colorectal cancer, focusing on the activation of the Nrf2 system. The animals presented elevated MDA levels and decreased SOD, CAT, and GSH antioxidant enzymes. The animals also showed elevated iNOS and COX-2. However, these oxidative and pro-inflammatory scenarios were reverted after cocoa treatment. The treatment increased Nrf2 and its downstream target expressions, such as NQO1 and UDP-GT. The authors highlighted these results as potent anticancer effects of cocoa, focusing on Nrf2 activation.

### 7.5. Tussilagone

Nam & Kim [171] studied the anticancer effects of tussilagone, a major component of *Tussilago farfara* L., in experimental colitis-associated colon cancer. The authors used the DSS-induced BALB/c mice model of colitis. The administration of tussilagone diminished the prevalence of colonic tumors among the animals, reduced cancer cell proliferation, induced cancer cell apoptosis, and decreased β-catenin expression. This bioactive compound also exerted antioxidant effects against tumor formation due to increased HO-1 expression.

### 7.6. Glucosinolates 

Lippmann et al. [172] showed inhibition of intestinal inflammation and reduced colon cancer differentiation in a model of inflammation-triggered colon carcinogenesis after using glucosinolates from pak choi and broccoli. The authors showed that a diet rich in these compounds up-regulated the expression of different sets of Nrf2 target genes such as Nqo1, Gstm1, Srxn1, and GPx2 and drastically reduced both colitis and tumor development.

### 7.7. Crocin

Kawabata et al. [173] studied the inhibitory effects of the carotenoid crocin against colitis and colitis-associated colorectal carcinogenesis using a DSS-induced ICR mice model of IBD. The results showed that dietary crocin inhibited NF-κB in the mice colon, as well as diminished the expression of TNF-α, IL-1β, IL-6, IFN-γ, iNOS, and COX-2 and increased Nrf2 expression. The authors suggested that the specific anti-inflammatory and antioxidant effects of Nrf2 were associated with reduced carcinogenesis prevalence histologically.

### 7.8. Peracetylated (−)-Epigallocatechin-3-Gallate (AcEGCG)

Chiou et al. [174] intended to evaluate the anticancer effects of AcEGCG against colon carcinogenesis in a DSS-induced mice model of colitis and colorectal cancer. The authors considered AcEGCG as a potent anti-colitis agent due to its effects on preventing the shortening of colon length and formation of aberrant crypts, as well as lymphoid nodules in the DSS-stimulated mice, and decreasing inflammation through down-regulating of Phosphoinositide 3-kinases (PI3K)/Akt /NF-κB phosphorylation and p65 acetylation. However, the most pronounced anticancer effect of AcEGCG was antioxidative, which augmented HO-1 expression via ERK1/2 signaling and acetylation of Nrf2.

### 7.9. Pterostilbene 

Chiou et al. [175] evaluated the effects of pterostilbene in colon carcinogenesis during AOM-induced colon tumorigenesis in BABL/c mice. The results showed that pterostilbene could significantly inhibit aberrant crypt foci disruption, lymphoid nodule formation, and tumor development. These results were accomplished due to the activation of the Nrf2 signaling pathway and the inhibition of NF-κB activation by blocking the phosphorylation of protein kinase c (PKC) β2 and decreasing downstream target gene expression, as well as inducing the blockage of iNOS, COX-2, and aldose reductase production, and augmenting HO-1 and glutathione reductase (GR) expression. When taken together, the results are related to the Nrf2 activation that blocks inflammation and OS through induction of HO-1 and GR expression, thereby preventing AOM-induced colon carcinogenesis during this model of IBD.

### 7.10. Nobiletin

Wu et al. [176] studied the effects of nobiletin (NOB), a major citrus polymethoxyflavone, in colitis-associated colon carcinogenesis using a DSS-induced mice model of IBD, LPS-stimulated macrophages, and human colon HCT116 cancer cells. The results demonstrated that NOB and its metabolites (NOB-Met) could effectively decrease the cells’ production of iNOS, increase HO-1, augment NQO1, and up-regulate the Nrf2 signaling pathway in mice and LPS-stimulated macrophages. Through principally acting on Nrf2, NOB and NOB-Met significantly promoted colon HCT116 cancer cell cycle arrest in human colon cancer cells by profoundly modulating key cyclins and cyclin-dependent kinases proteins.

### 7.11. Wogonin

The effects of wogonin were investigated by Yao et al. [177] in colorectal carcinogenesis. The authors found that wogonin lowered tumor incidence and inhibited colorectal adenoma development in a DSS-induced mice model of IBD-associated carcinogenesis. Wogonin also decreased the secretion and expression of IL-6 and IL-1β, reduced cell proliferation and augmented Nrf2 expression and nuclear translocation in adenomas and surrounding tissues, and decreased the inflammatory pathway expression, such as NF-κB. While experimenting with colon cancer HCT116 cells, the authors found that wogonin effectively inhibited the interaction between human monocytic THP-1 cells and human colon cancer HCT116 cells. Moreover, it effectively inhibited LPS-induced THP-1 cells from prototypically expressing and secreting the pro-inflammatory cytokines IL-6 and IL-1β. Although many results were described, further mechanistic research revealed that wogonin inhibited colorectal tumor development by regulating Nrf2 activation and signaling and decreasing the nuclear translocation of NF-κB and phosphorylation of IkB and IKKα/β in HCT116 cells and THP-1 cells.

### 7.12. Cinnamaldehyde

Long et al. [178] investigated the effects of cinnamaldehyde (derived from cinnamon) in the chemoprevention against colorectal cancer in colorectal epithelial cells of a colorectal cancer model comparing Nrf2+/+ with Nrf2−/− mice. In vitro, the authors found that cinnamaldehyde caused a significant Keap1-C151-dependent increase in Nrf2 protein expression and half-life by the treated HCT116 cells. The results were accomplished via the blockage of ubiquitination with upregulation of Nrf2 cytoprotective target genes and elevation of cellular glutathione. In vivo, cinnamaldehyde supplementation also suppressed colon carcinogenesis in mice, and the suppression was confirmed in Nrf2+/+ but not in Nrf2−/− mice.

## 8. Future Perspectives

### 8.1. Algae-Derived Constituents in Regulating Nrf2 in IBD Models 

Bagalagem et al. [179] explored a natural compound derived from brown seaweed, a sulfated polysaccharide known as fucoidan. This study used AA-induced Sprague Dawley rats, and some were treated with fucoidan. The induced rats presented a significant reduction in colon length and elevation in colon weight. However, when fucoidan treatment was initiated, these findings were reverted. Fucoidan also elevates Aryl hydrocarbon receptor, which is reduced in patients with UC and is associated with IL-22, an anti-inflammatory organokine that, when increased, improves the epithelial barrier and the microbiota and increases mucosal healing. Other benefits of fucoidan in this study were the increased expression of cyclic adenosine monophosphate (cAMP), Nrf2, and HO-1, in addition to reduced OS. 

Ardizzone et al. [180] studied the effects of a marine green seaweed rich in polyunsaturated fatty acids, *Ulva pertusa*, against Nrf2 deactivation in IBD. The treatment was carried out in the DNBS-induced CD1 mice model of colitis. *Ulva pertusa* showed immunomodulatory and anti-inflammatory properties by upregulating Nrf2 and inducing HO-1 activity. It also reduced COX-2 and iNOS and modulated the NF-κB pathway during the inflammatory burden of the animals’ intestines during IBD development. This seaweed also restored the damage caused by induced colitis, improved tissue restoration, reduced intestine edema, and reversed weight loss. 

### 8.2. Fungus-Derived Constituents Regulating Nrf2 in IBD Models

Impellizzeri et al. [181] used a DNBS-induced CD1 mice model of colitis to evaluate the effects of the fungus *Coriolus versicolor* in a dose of 200 mg/kg against IBD. Before treatment, they observed neutrophils infiltration in the colon, lipid peroxidation, and increased pro-inflammatory cytokines secretion, such as IL-1β and TNF-α. *Coriolus versicolor* decreased the expression of TLR4 and reduced the NF-κB pathway activation and signaling, reverting the inflammatory and oxidative previous scenario. These actions were principally related to the Nrf2 activation and consequent HO-1 expression.

Gao et al. [182] studied *Saccharomyces boulardii*, a probiotic yeast resistant to low pH and highly tolerant to bile acids, in mice models of UC by regulating the NF-κB and Nrf2 signaling pathways. The authors used a DSS-induced C57BL/6 mice model of colitis and, after treatment with 105 and 107 CFU/mL, p.o. of *S. boulardi*, this probiotic reduced DAI effects and reestablished the gut health of the treated animals. Significantly, it also prevented IκBα degradation, suppressed nuclear translocation of NF-κB, reduced the activity of MPO, regulated inflammatory cytokines, and acted as an immune modulator. These actions were principally related to the Nrf2 signaling activation and the transcription of genes of antioxidant enzymes such as HO-1.

Li et al. [183] explored *Gloeostereum incarnatum*, a fungus rich in amino and fatty acids, on UC via modulation of Nrf2/NF-κB signaling in C57BL/6 mice. The use of the fungus reduced ROS and pro-inflammatory cytokines, such as IL-1β, IL-2, IL-6, IL-12, TNF-α, TNF-β, IFN-α, and IFN-γ. It also reduced the phosphorylated activation of NF-κB and regulated Nrf2/NF-κB signaling by principally decreasing CAT, HO-1, SOD-1, and SOD-2.

### 8.3. Bee Pollen Regulating Nrf2 in IBD Models

Bee pollen has polyphenolic compounds and provides various health benefits, mainly by its anti-inflammatory properties. Li et al. [184] studied bee pollen, formed with plant pollens mixed with nectar and bee secretions, to evaluate its effects on the Caco-2 intestinal barrier dysfunction induced by a DSS model. After treatment, they observed higher expressions of Nrf2, Txnrd1, and NQO1. It also blocked the MAPK signaling pathway and significantly reduced OS and inflammation by promoting TGF-β1 gene expression and suppressing TNF-α and IL-6.

### 8.4. Thai Royal Jelly Regulating Nrf2 in IBD Models

Jenkhetkan et al. [185] explored Thai royal jelly (TRJ), a milky-acid secretion produced by worker bees that feed the queen bees, in a model of IBD-associated inflammation. The study was carried out using a human lymphocytes-enriched buffy coat in vitro treated with TRJ (0.0005–5 mg/mL). During the study, the authors observed that the cells presented increased antioxidant action through the up-regulation of Nrf2 and HO-1 antioxidant elements. These findings suggested that TRJ can be helpful against colitis.

## 9. Conclusions

Numerous plants and phytochemicals can be used as therapeutic adjuvants in IBD and colorectal cancer associated with IBD since they can regulate Nrf2 and, therefore, among many actions, reduce inflammation and OS and increase apoptosis of cancer cells. These effects improve the bowel environment, mucosal barrier, colon, and crypt disruption, reduce ulceration and microbial translocation, and consequently reduce the disease activity index. Moreover, the modulation of Nrf2 can regulate various genes involved in cellular redox, protein degradation, DNA repair, xenobiotic metabolism, and apoptosis, contributing to the prevention of colorectal cancer.

Even though only in vivo and in vitro studies were found in the consulted databases, our results bring to light the need for clinical trials so that the effects on human beings can be investigated and new paths can be paved in the search for effective therapy (or effective adjuvant therapy) for IBDs, whose incidence increases rapidly and drastically reduces the quality of life and capacity for work and social interaction of the carriers.

As a limitation, our study is not different from other narrative reviews. Due to the significant number of included studies, determining and integrating complex interactions between different models of IBD and IBD-associated colorectal cancer presented to be a challenge. However, this could be overcome by the objective of this review, which was to respond to the question, “What are the effects of medicinal plants and natural and synthesized phytochemicals as regulators of the Nrf2 pathways in IBD and colitis-associated colorectal cancer?’’ and giving to light the need for clinical trials.

The present review is the first in the medical literature to evaluate the roles of medicinal plants and natural and synthesized phytochemicals in Nrf2 regulation during IBD and IBD-related colorectal cancer. In addition, to our knowledge, Nrf2 regulation in IBD and IBD-associated carcinogenesis was never before revised in light of a narrative review that organized the databases’ model studies in tables with our included topics.

## Figures and Tables

**Table 1:** Studies regarding the use of medicinal plants with regulatory effects on Nrf2 signaling pathway in IBDs.

Medicinal Plants	In Vivo/In Vitro Models	Plant Parts Used or Extracts	Effective Doses/Concentrations	Related Clinical Features of IBD	Nrf2-Related Dysregulation Indicators	Related Molecular Mechanisms in Nrf2 Regulation in IBD	Ref.
*Cynara cardunculus* L. Asteraceae	TNF-α-induced intestinal Caco-2 epithelial cells model of colitis	Leaves extract rich in chlorogenic acid and luteolin	5, 10, and 15 μg/mL	↑Intestinal inflammation and ↑OS	↑NF-κB activation, ↑ROS intracellular levels, ↓TAA, and ↓GSH	↑Nrf2 nuclear translocation, ↑GCLC mRNA, and ↑NQO1 mRNA	[17]
*Panax ginseng* Araliaceae	DSS-induced C57BL/6J mice model of colitis and LPS-treated RAW264.7 cells	Root extract rich in ginsenosides	32.5, 125, and 500 μg/mL	↑Intestinal inflammation and ↑OS	↑NO, ↑TNF-α, ↑IL-6, ↑IL-1β, ↓IL-10, ↑ROS, and ↑mitochondrial dysfunction	↑Nrf2 nuclear translocation, ↑p62 phosphorylation, and ↑Akt-mTOR signaling	[18]
*Rose odorata sweet* var. *gigantean* (Coll. et Hemsl.) Rehd. et Wils Rosaceae	DSS-induced C57BL/6 mice model of colitis	Root extract rich in total tannins and triterpenoids	125, 250 and 500 mg/kg	↑DAI, ↑OS, ↑loss of epithelial and goblet cells, ↑crypt aberrations, and ↑prominent transmural inflammatory cells infiltration	↑NOS, ↑MDA, ↑MPO, ↑TNF-α, ↑IL-6, and ↑IL-1β	↑HO-1, ↑SOD, ↓number of nuclear NF-κB-p65 positive cells, ↓p-NF-κB-p65, ↓p-IKKα/β, and ↓Keap1	[19]
*Ficus pandurata* Hance Moraceae	DSS-induced C57BL/6 mice model of colitis	Crude extract	24-48 g/kg	↑DAI, ↑intestinal inflammation, and ↑OS	↑NF-κB activation, ↓intestinal barrier integrity, ↑MPO, ↓DAO, ↓T-SOD, ↓GSH-Px, ↑MDA, and ↓HO-1	↓TLR4/MyD88/NF-κB, ↑DAO, ↑T-SOD, ↓Keap1, ↓NOX2, ↓p22-phox, and ↑Nrf2 translocation, ↑HO-1, and ↑NQO1	[20]
*Moringa oleifera* Lam. Moringaceae	DSS-induced C57BL/6 mice model of colitis	Isothiocyanate-enriched seed extract	150 mg/kg	↑DAI, ↓intestinal integrity, ↑intestinal inflammation, and ↑OS	↑TNF-α, ↑IL-1β, ↑IL-6, ↑NO, ↑iNOS, and ↑MPO	↓Pro-inflammatory genes expression, ↑GSTP1, ↑NQO1, ↑HO-1, and ↓NF-κB-dependent pro-inflammatory signals	[21]
*Aucklandia lappa* Decne. Asteraceae	DSS-induced C57BL/6 mice model of colitis and LPS-treated RAW264.7 cells	Aqueous and SLRF (rich in COS and DHL) ALDE extracts	1.82 g/kg for aqueous or 0.91 g/kg-1.82 g/kg for SLRF extracts in mice and 2, 4, or 8 μM for COS or 1.25, 2.5, or 5 μM for DHL in treated cells	↑DAI and ↑intestinal inflammation	↑TNF-α, ↑IL-1β, ↑IL-6, ↑M1 macrophage polarization, ↑iNOS, ↑COX-2, ↑NF-κB-related proteins, ↓HMOX-1, ↑p38 MAPK phosphorylation, ↑NF-κB p65 phosphorylation, ↑Erk phosphorylation, and ↑JNK phosphorylation	↓IL-1β mRNA, ↓IL-6 mRNA, ↓TNF-α mRNA, ↓MAPK expression, ↓NF-κB expression, ↑HMOX-1 mRNA expression, ↑Nrf2 mRNA expression in mice, and ↓p38MAPK phosphorylation, ↓p-NF-κB-p65 phosphorylation, ↓Erk phosphorylation, ↓JNK phosphorylation, ↑Nrf2 expression, and ↑HMOX-1 expression in treated cells	[22]
*Vaccinium myrtillus* L. Ericaceae and *Ribes nigrum* L. Grossulariacae	Caco-2 human intestinal epithelial cells exposed to TNF-α	ACN-rich purified and standardized *Vaccinium myrtillus* and *Ribes nigrum* extract	0.18, 0.37, 0.75, and 1.5 µg/mL	↑Intestinal inflammation	↑TNF-α-induced nuclear translocation of NF-κB-p65, ↑IL-8 mRNA, and ↑IL-6 mRNA,	↓NF-κB-p65 subunit translocation, ↑Nrf2 nuclear translocation, and ↑NQO1 mRNA	[23]
*Mesua assamica (King&Prain)* Kosterm Calophyllacae	NF-κB-RE-luc2P HEK 293 TNF-α-stimulated cells and DSS-induced C57BL/6 mice model of colitis	Bark ethanolic extract	100 and 200 mg/kg	↑Intestinal inflammation and ↑OS in vitro and ↑DAI, ↓intestinal integrity, splenomegaly, and ↓colon length in vivo	↑MDA, ↑MPO, ↑nitrite levels, ↓GSH, ↑inflammatory cells accumulation, ↑IL-6, ↑IL-1β, and ↑TNF-α,	↓NF-κB-related proteins expression, ↓STAT3 signaling cascades, ↓IKBα phosphorylation, ↑SOD2, ↑HO-1, and ↑SIRT1	[24]
*Vaccinium myrtillus* L. Ericaceae	DSS-induced *Drosophila melanogaster* model of intestinal inflammatory damage	Berry anthocyanin extracts	0.1 mg/mL	↓Intestinal integrity, ↓intestinal length, ↑intestinal inflammation, and ↑intestinal OS	↑ROS content and ↑pro-inflammatory cytokines levels	↑Nrf2-related protein genetic expression (↑GCL, ↑GSTS, and ↑SOD)	[25]
*Vitis vinifera* L. Vitaceae	DSS-induced C57BL/6 mice model of colitis	Seed proanthocyanidin extract	-	↓Body weight, ↑diarrhea, and ↑bloody stool	↑IL-1β, ↑IL-6, ↑TNF-α, ↑NO, ↑MDA, ↓SOD, ↑NF-κB-related proteins expression, and ↓HO-1	↑HO-1 and other Nrf2-related protein’s genetic expression, and ↓NF-κB-related proteins expression	[26]
*Acanthopanax senticosus* (Rupr. et Maxim) Araliaceae	H2O2-Induced RAW 264.7 Cells and DSS-induced C57BL/6 mice model of colitis	Flavonoid extract	15, 30, 60, 90, 120, and 150 mg/L in treated cells and 50, 100, and 200 g/L in treated mice	↑Intestinal inflammation and ↑OS in vitro and ↑DAI in vivo	↑MDA, ↓CAT, ↓SOD, ↓GPx, ↑Keap1, and ↓HO-1	↓Keap1 and ↑HO-1	[27]
*Forsythia suspensa* (Thunb.) Vahl Oleracea	DSS-induced C57BL/6 mice model of colitis	Polyphenol-rich extract	0.1, 0.2, and 0.4 g/mL	↑DAI, ↓body weight, ↑intestinal villi degeneration, ↑necrosis, ↑proliferation and infiltration of inflammatory cells in the intestine	↑IL-1β, ↑MDA, ↓SOD, and ↑MPO	↓MDA, ↑SOD, ↓MPO, ↑Nrf2 activity, ↑HO-1, ↑NQO1, ↓caspase-1 expression, ↓IL-1β expression, ↓GSDMD expression, ↓NLRP3 expression, ↓ROS levels, ↓pyroptosis, and ↓ASC	[28]
*Artemisia argyi* H. Lév. & Vaniot Asteraceae	DSS-induced C57BL/6 mice model of colitis	Ethanolic extract	200 mg/kg	↑DAI, ↑colonic dysplasia, ↑colon barrier disruption, and ↓body weight	↑AST, ↑ALT, ↑IL-6, ↑IL-1β, ↑TNF-α, and ↑COX-2	↓IL-6, ↓IL-1β and ↓TNF-α expressions, ↓p-IκBα, ↓p-NF-kB, ↓COX-2 expression, ↓ICAM-1 expression, ↓MCP1 expression, ↓iNOS expression, ↑Nrf2 activity, and ↑HO-1	[29]
*Dendrobium fimbriatum* L. Orchidaceae	DSS-induced mice model of colitis	Polysaccharide extract	-	↑DAI, ↑colon barrier disruption, and ↓body weight	↑NF-κB signaling and ↑Th17/regulatory T homeostasis disruption	↓NF-κB signaling activation, ↓Th17/regulatory T homeostasis disruption, and ↑Nrf2 signaling	[30]
*Pisum sativum* L. Fabaceae	DSS-induced C57BL/6 mice model of colitis	Hull polyphenol extracts	100 and 600 mg/kg	↑DAI, ↑diarrhea, blood in the stool, ↓colon length, and ↓body weight	↑MPO, ↓claudin-1, ↓occludin, ↓ZO-1, ↑MDA, ↓SOD, ↓CAT, ↓T-AOC, ↑TNF-α, ↑IL-1β, ↑IL-6, ↓IL-10, ↑Keap1, ↓Nrf2 expression, ↓GCLC, ↓HO-1, and ↓NQO1	↓MPO expression, ↑claudin-1 expression, ↑occludin expression, ↑ZO-1 expression, ↓MDA, ↑SOD, ↑CAT, ↑T-AOC, ↓TNF-α expression, ↓IL-1β expression, ↓IL-6 expression, ↑IL-10 expression, ↓Keap1, ↑Nrf2 expression, ↑Nrf2 nuclear translocation, ↑GCLC, ↑HO-1, and ↑NQO1	[31]
*Tetrastigma hemsleyanum* Diels & Gilg Vitaceae	DSS-induced mice model of colitis	Leaves bioactive and their metabolites	-	↓Colon length and ↓body weight	↓Claudin-1, ↓occludin, ↓ZO-1, ↑IL-1β, ↑IL-6, ↑TNF-α, ↓IL-10, ↓Nrf2 nuclear translocation, ↓SOD, ↓CAT, ↓HO-1, ↓NQO1, ↓GCLC, ↑MPO, and ↑MDA	↑Claudin-1, occludin and ZO-1 expressions, ↓IL-1β, IL-6 and TNF-α expressions, ↑IL-10 expression, ↑Nrf2 nuclear translocation, ↑SOD, ↑CAT, ↑HO-1, ↑NQO1 and ↑GCLC expression, ↓MPO, and ↓MDA expressions	[32]
*Rhus chinensis* Mill. Anacardiaceae	DSS-induced mice model of colitis	Fruits ethanolic extrat	-	↑DAI, ↑colon barrier disruption, and ↓colon length	↑MPO, ↑MDA, ↑IL-1β, ↑IL-6, ↑TNF-α, ↑p-NF-κB, ↑p-IκB, ↑COX-2, ↑iNOS, ↑p-P38, ↑p-Erk1/2, ↑p-JNK, ↓SOD, ↓GSH, ↓claudin-1, ↓occludin, ↓ZO-1, ↓Nrf2 activation, ↓NQO1, and ↓HO-1	↓MPO, ↓MDA, ↓IL-1β, ↓IL-6, ↓TNF-α, ↓p-NF-κB, ↓p-IκB, ↓COX-2, ↓iNOS, ↓p-P38, ↓p-Erk1/2 and ↓p-JNK expressions, ↑SOD, ↑GSH, ↑claudin-1, ↑occludin and ↑ZO-1 expressions, ↑Nrf2 activation and ↑NQO1, and ↑HO-1 expressions	[33]
*Crocus sativus* L. Iridaceae	DSS-induced C57BL/6 mice model of colitis	Aqueous extract	7.5, 15, 20, and 25 mg/kg	↑DAI, ↓colon length, and ↓body weight	↓HO-1, ↓GPX-2, ↓Nrf2 expression and activity, ↑TNF-α, and ↑IFN-γ	↑HO-1, ↑GPX-2, ↑Nrf2 expression and activity, ↓TNF-α and ↓IFN-γ expressions	[34]
*Prunus mahaleb* L. Rosaceae	DSS-induced BALB/c mice model of colitis	Concentrated fruit extract	1300 mg/Kg	↑Intestinal inflammation and ↑OS	↓Nrf2 expression and activity, ↓GSH, ↓GSR, and ↓G6PD	↑Nrf2 expression and activity, ↑GSH, ↑GSR, and ↑G6PD	[35]
*Quercus ilex* L. Fagaceae	TNBS-induced Wistar mice model of colitis	Leaves polyphenolic extract	250 and 500 mg/kg	↑Anorexia, ↓body weight, ↑intestinal adhesions to adjacent organs, ↑weight/length ratio of the colon, ↑transmural necrosis, ↑edema, ↑diffuse inflammatory cells infiltration, ↑ulceration, and ↑crypt distortion	↑MPO, ↑TNF-α, ↑IL-1β, ↑COX-2, ↑iNOS, ↑IκB-α degradation, ↑p65 nuclear translocation, and ↑p50 nuclear translocation, ↑NF-κB-mediated transcriptional protein activation, ↑MAPK (JNK, p38, and ERK1/2) protein activation, ↓HO-1, and ↓Nrf2-mediated transcriptional protein activation	↓MPO, ↓TNF-α, ↓IL-1β, ↓COX-2 and ↓iNOS expressions, ↓IκB-α degradation, ↓p65 and ↓p50 nuclear translocation, ↓NF-κB-mediated transcriptional protein activation, ↓MAPK (JNK, p38, and ERK1/2) protein activation, ↑HO-1 expression, and ↑Nrf2-mediated transcriptional protein activation	[36]
*Ziziphus spina-christi* L. Rhaminaceae	AcOH-induced Wistar mice model of colitis	Fruits extract	100, 200, and 400 mg/kg	↑Weight/length ratio of the colon, ↓mucin concentration, ↑necrosis ↑ulceration, ↑corrosion, ↑hemorrhagic diarrhea, ↑intestinal focal infiltration of inflammatory cells, and ↑crypt distortion	↑MPO, ↑LPO, ↑NO, ↓GSH, ↓SOD, ↓CAT, ↓GSR, ↓GPx, ↑TNF-α, ↑IL-1β, ↑iNOS, ↑COX-2, ↑NF-κB activation and signaling, and ↓Nrf2/HO-1 activation and signaling	↓p38, ↓MAPK, and ↓VEGF-A expressions, ↓Bax and ↓caspase-3-mediated apoptosis, ↑Bcl-2 expression, ↓MPO, ↓LPO, and ↓NO expressions, ↑GSH, ↑SOD, ↑CAT, ↑GSR, and ↑GPx expressions, ↓TNF-α, ↓IL-1β, ↓iNOS, ↓COX-2 expressions, ↓NF-κB activation, and signaling and ↑Nrf2/HO-1 activation, and signaling	[37]
*Perilla frutescens* L. Laminaceae	DSS-induced ICR mice model of colitis	Leaf extracts	20 and 100 mg/kg	↓Body weight, ↑DAI, ↑diarrhea, ↑bloody stool, ↓colon length, and ↑intestinal infiltration of inflammatory cells	↑COX-2, ↑iNOS, ↑cyclin D1, ↑NF-κB activation and translocation, ↑IκBα phosphorylation and degradation, ↑p65 nuclear translocation, ↑STAT3 signaling, ↑TLR4, ↑IRF3, ↑IFN-α, ↑IFN-γ, ↓Nrf2/HO-1 activation and signaling, and ↓HO-1	↓COX-2, ↓iNOS and ↓cyclin D1 expressions, ↓NF-κB activation and translocation, ↓IκBα phosphorylation and degradation, ↓p65 nuclear translocation, ↓STAT3 signaling, ↓TLR4, ↓IRF3, ↓IFN-α, and ↓IFN-γ expressions, ↑Nrf2/HO-1 activation and signaling, and ↑HO-1	[38]

↑, increase; ↓, decrease; AcOH, acetic acid; Akt-mTOR, protein kinase b-mammalian target of rapamycin; ALDE, *Aucklandia lappa* Decne.; ALT, alanine aminotransferase; ANC, anthocyanins; ASC, apoptosis-associated speck-like protein; AST, aspartate transaminase; Bax, BCL2 associated x, apoptosis regulator; Bcl-2, B-cell lymphoma 2; COS, costunolide; COX-2, cyclooxygenase; DAO, diamine oxidase activity; DAI, disease activity index; DSS, dextran sulfate sodium; DHL, dehydrocostus lactone; Erk, extracellular signal-regulated kinase; G6PD, glucose-6-phosphate dehydrogenase; GCL, glutamate-cysteine ligase; GCLC, catalytic glutamate-cysteine ligase; GPX-2, glutathione peroxidase 2; GSDMD, gasdermin D; GSH, glutathione; GSH-Px, glutathione peroxidase; GSR, glutathione reductase; GSTP1, glutathione S-transferase P; GSTS, glutathione S-transferases; HMOX-1, heme oxygenase 1 gene; HO-1, heme oxygenase 1; ICAM-1, intercellular adhesion molecule 1; IL-1β, interleukin 1 beta; IL-6, interleukin 6; IL-10, interleukin 10; iNOS, inducible nitric oxide synthase; JNK, c-Jun N-terminal kinase; Keap1, Kelch-like ECH associated protein 1; LPO, lipid peroxidation; LPS, lipopolysaccharide; MAPK, mitogen-activated protein kinase; MCP-1, monocyte chemoattractant protein-1; MDA, malondialdehyde; mRNA, messenger RNA; MPO, myeloperoxidase; NF-κB, nuclear factor kappa b; NF-κB-p65, NF-κB transcription factor p65; NLRP3, NLR family pyrin domain containing 3; NO, nitric oxide; NOS, nitric oxidase synthase; NOX-2, NADPH (nicotinamide adenine dinucleotide phosphate hydrogen) oxidase 2; NQO1, NAD(P)H quinone oxidoreductase-1; Nrf2, nuclear factor erythroid 2-related factor 2; OS, OS; p-IκBα, nuclear factor of kappa light polypeptide gene enhancer in B-cells inhibitor, alpha; p-IKKα/β, IKK serine kinase proteins α/β; p-NF-κB-p65, NF-κB transcription protein factor 65; NQO1, quinone oxidoreductase 1; p22-phox, human neutrophil cytochrome b light chain; ROS, reactive oxygen species; SLRF, sesquiterpene lactone-rich fraction; SOD, superoxide dismutase; STAT3, signal transducer and activator of transcription 3; TAA, total intracellular antioxidant capacity; T-AOC, total antioxidant capacity; Th17, T helper 17; TLR4/MyD88/NF-κB, toll-like receptor/myeloid differentiation primary response 88/nuclear factor kappa b; TNBS, trinitrobenzene sulfonic acid; TNF-α, tumor factor necrosis alfa; T-SOD, total superoxide dismutase; VEGF-A, vascular endothelial growth factor a.

**Table 2:** List of natural and synthesized phytochemicals with regulatory effects on (Nrf2) signaling pathways in IBD.

Compounds/Phytochemicals	In Vivo/In Vitro Model	Effective Doses/Concentrations	Related Clinical Features of IBD	Nrf2-*Related Dysregulation Indicators*	Related Molecular Mechanisms in the Regulation of Nrf2	Ref.
6-shogaol ** 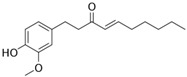 **	Raw 264.7 macrophage and colon-26 in vitro LPS-activated cells and DSS-induced FVB/NJ mice model of colitis in vivo	50, 100, 200, 500, and 1000 µg/mL in vitro and 15 mg/kg in vivo	↑Intestinal inflammation and ↑OS both in vitro and in vivo and ↓intestinal integrity and ↑intestinal leukocyte invasion in vivo	↑Lcn-2, ↑TNF-α, ↑IL-6, and ↑IL-1β	↑HO-1, ↓TNF-α mRNA, ↓IL-6 mRNA, ↓IL-1β mRNA, and ↓iNOS mRNA,	[39]
Licochalcone A ** 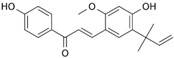 **	DSS-induced C57BL/6 mice model of colitis	20, 40, and 80 mg/kg	↓Colon length, ↓stool consistency, ↑colonic necrosis, ↑inflammatory cells infiltration, and thickening of muscular mucosa	↑MPO, ↑TNF-α, ↑IL-6, ↑IL-1β, ↑COX-2, ↑NF-κB p65, ↑IKKα, ↑p-IκB, ↑NO, ↓SOD, ↓GSH, and ↑Keap1	↑HO-1 and ↑GCL expressions and ↓Keap1, ↓NF-κB p65, ↓IKKα, and ↓p-IκB expressions	[40]
	DSS-Induced C57BL/6 mice model colitis	20, 40, and 80 mg/kg	↑Intestinal inflammation and ↑OS	↑TNF-α, ↑IL-6, ↑IL-1β, ↑COX-2, and ↑ROS	↓p-NF-κB-p65, ↓pIkB kinase α, ↑HO-1, ↑GCLC, ↓Keap1, and ↑Nrf2 translocation and signaling	[40]
Oligonol ** 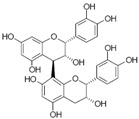 **	DSS-induced C57BL/6 mice model colitis	10, 50, and 100 mg/kg	↑Intestinal inflammation, ↑OS, ↑destruction of the mucosal barrier, and ↓colon length	↑TNF-α, ↑IL-6, ↑IL1β, ↑COX- 2, ↑iNOS, ↑ROS, ↑JNK, and ↓HO-1	↓NF-κB-p65, ↑Nrf2 transcription and signaling, ↑HO-1, ↑AKRs, and ↑ GSTs	[41]
Cyanidin-3-O-glucoside ** 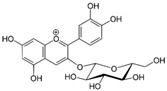 **	TNF-α-induced intestinal Caco-2 epithelial cells	20-40 µM	↑Intestinal inflammation, ↑OS, and ↑cellular damage	↑TNF-α, ↑IL-6, ↑COX- 2, ↑PGE2, ↑thromboxane A2, ↑leukotrienes, ↑ROS, and ↓ GSH	↓NF-κB-p65, ↓NF-κB-p50, ↑Nrf2 expression and signaling, ↑NQO1, and ↑HO-1	[42]
Luteolin ** 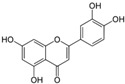 **	DSS-induced C57BL/6 mice model colitis	20 and 50 mg/kg	↑Intestinal inflammation, ↑OS, ↑glands destruction, and ↑colon length	↑TNF-α, ↑IL-6, ↑iNOS, and ↑MDA	↓NF-κB-p65, ↑Nrf2 expression and signaling, ↑NQO1, ↑HO-1 and ↑SOD, and ↑CAT expressions	[43]
Alpinetin ** 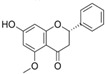 **	DSS-induced C57BL/6 mice model colitis	25, 50, and 100 mg/kg	↑DAI, ↑intestinal inflammation, ↑OS, ↓colon length, ↑loss of epithelial and goblet cells, and ↑crypt aberrations	↓SOD, ↓Nrf2 expression, and ↓HO-1	↑SOD expression, ↑Nrf2 expression and signaling, and ↑HO-1	[44]
Cardamonin 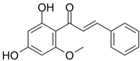	DSS-induced C57BL/6 mice model of colitis and TNBS-induced BALB/c mice model of colitis	15, 30, 60 mg/kg (DSS), and 200mg/kg (TNBS)	↑DAI, ↑OS, ↑distorted crypts, ↑loss of goblet cells, and ↑mucosal damage	↑TNF-α, ↑IL-6, ↑IL1β, ↑ NLRP3, ↑ROS, and ↑cleaved caspase-1	↑ Nrf2 transcription and signaling, ↑NQO1, ↑SOD expression, and↑ HO-1	[45]
Puerarin ** 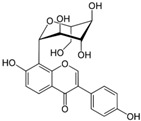 **	DSS-induced BALB/c mice model of colitis	10 and 50 mg/kg	↑DAI, ↑intestinal inflammation, ↑OS, ↑ colon length, ↑ spleen index, and ↑crypt aberrations	↑MPO, ↑TNF-α, ↑IL-6, ↑ IFN- γ, ↑IL-1β, ↑ROS, ↑COX-2, ↑PGE2 ↑iNOS, ↑MDA, ↓GSH, and ↓ ZO-1	↓p-NF-κB, ↑Nrf2 transcription, ↑NQO1, ↑ HO-1, ↑SOD, and ↑CAT expressions	[46]
Gallic acid ** 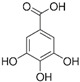 **	DSS-induced BALB/c mice model of colitis	10 mg/kg	↑OS, ↑intestinal inflammation, ↑DAI and ↑colon length, and ↑gland distortion	↑IL-23, ↑IL-21, and ↑MDA	↑SOD, ↑CAT, ↑Nrf2 expression and signaling, ↑NQO1, and ↑UDP-GT	[47]
Sulforaphane ** 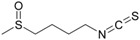 **	DSS-induced mice model of colitis	10, 15, and 20 mg/kg	↑Intestinal inflammation, ↑OS, and ↑barrier injury	↑TFAM expression, ↑mTOR expression, and ↑cyclin D1 expression	↑Nrf2 expression and signaling and ↑HO-1	[48]
	DSS-induced Cc57bl/6 mice model of colitis	2.5, 5, 10, and 20 mg/kg	↑OS, ↑intestinal inflammation, ↑DAI, and ↑ colon length	↑TNF-α, ↑IFN- γ, ↑ IL-1β, and ↑COX-2	↑Nrf2 expression and signaling	[49]
Asperuloside ** 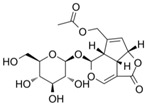 **	DSS-induced KM mice model of colitis and LPS-treated RAW264.7 cells	125 and 500 μg/kg in vivo and 5, 10, 20 μM in vitro	↑DAI, ↑inflammatory cell infiltration ↑OS, ↑colon length, ↑loss of epithelial and ↓goblet cells in vivo and ↑inflammation and ↑OS in vitro	↑MPO, ↓GSH, ↑ MDA, ↑TNF-α, ↑IL-6, ↓ IL-10, and ↑ROS	↓p-NF-ΚB-p65, ↑SOD, ↑Nrf2 expression, ↑NQO1 mRNA, and ↑HO-1 mRNA	[50]
Syringin ** 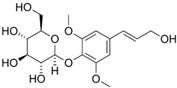 **	DSS-induced BALB/c mice model of colitis	100 mg/kg	↑Inflammatory cell infiltration, ↑OS, and ↑colon length	↑TNF-α, ↑IL-6, ↑IL1β, ↑ COX-2, ↑iNOS, ↓ZO-1, and ↓occludin	↓p-NF-κB, ↓IκBα, ↓p-IκBα, ↑Nrf2 expression, and ↑HO-1	[51]
Paeoniflorin ** 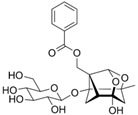 **	LPS-treated Caco-2 cells	10, 50, 100, and 150 μM	↑Intestinal inflammation, ↑OS, and ↑loss of mucosal barrier proteins	↑TNF-α, ↑IL-6, ↑COX-2, ↑iNOS, ↓ZO-1, and ↓occludin	↓p-NF-κB, ↑Nrf2 mRNA expression, and ↑HO-1	[52]
Dehydrocostus lactone ** 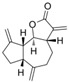 **	DSS-induced ICR mice model of colitis and LPS-treated RAW264.7 cells	5–15 mg/kg in vivo and 2–9 μM in vitro	↑Intestinal inflammation, ↑OS, ↑barrier injury, and ↓goblet cells in vivo and ↑Intestinal inflammation and ↑OS in vitro	↑MPO, ↑TNF-α, ↑IL-6, ↑ COX-2, and ↑ROS	↓p-NF-κB p-65, ↑IKKα/β, ↑Nrf2, ↑ HO-1, ↓Keap1, and ↓MAPK	[53]
Leonurine ** 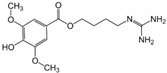 **	DSS-induced C57BL mice model of colitis	7, 5–15, and 30 mg/kg	↑DAI, ↑inflammatory cell infiltration, ↑OS, and ↓colon length	↑TNF-α, ↑IL-6, ↑IL-1β, and ↓GSH	↑Nrf2 expression, ↑ HO-1, ↓p-NF-κB, ↓TLR4, and ↑SOD	[54]
Crocin ** 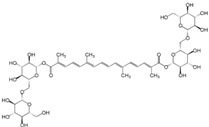 **	AA-induced Sprague Dawley mice model of colitis	20 mg/kg	↑DAI, ↑stromal edema, ↑ulcerated mucosa, ↑infiltration of inflammatory cells, and ↑lymphoid follicles	↓TAC, ↓CAT, ↓SOD, ↓GSH, ↑MDA, ↑TNF-α, ↓Nrf2, ↓ HO-1, ↑ROS, and ↑apoptosis	↓TNF-α expression, ↓ROS production, ↓caspase-3 expression, ↓MDA, ↑Nrf2, ↑HO-1, ↑, ↑CAT, ↑SOD, and ↑GSH expression	[55]
Quercetin ** 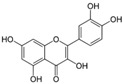 **	DSS-induced Wistar mice model of colitis	10, 15, and 20 mg/kg	↑DAI, ↑coagulative epithelial necrosis, ↑intestinal inflammation, ↑inflammatory cell infiltration, ↓colonic crypts, ↑ulceration in epithelial tissues, ↓mucin, and ↓goblet cells	↑MPO, ↑IL-6, ↑TNF-α, ↑ IFN-γ, ↑IL-10, ↑COX-2, ↑MDA, ↑ROS, ↑NF-κB, ↑CD-4, ↑CD-8, ↑TLR-4, ↑inflammatory cytokines, ↑proteolytic enzymes, ↓MUC2 genetic expression, ↓occludin, ↓plasma antioxidants, ↓GSH, ↓SOD, and ↓CAT	↑Nrf2 and HO-1 genetic expression, ↑occludin, ↑MUC2 genetic expression, ↓NF-κB activation, and signaling	[56]
8-Oxypalmatine ** 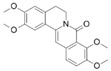 **	DSS-induced Balb/c mice model of colitis	12, 5, 25, and 50 mg/kg	↑ DAI, ↑markers of OS, ↑inflammatory mediators ↓body weight, ↑colon shortening, ↑massive infiltration of inflammatory cells, ↑excessive crypt damage, ↑epithelial cell destruction, and ↑mucosal thickening	↑NLRP3, ↑MPO, ↑TNF-α, ↑IL-1β, ↑IFN-γ, ↑IL-17, ↑IL-6, ↑NO, ↑MDA, ↑ASC, ↑caspase-1, ↓SOD, ↓GSH, ↓CAT, ↓GSH-Px, ↓Nrf2 expression, ↓HO-1, and ↓IL-10	↓NLRP3 activation and signaling, ↓ASC expression, ↓caspase-1, ↑Nrf2 expression and ↑HO-1, ↓pro-inflammatory cytokines expression, and ↑anti-inflammatory cytokines expression	[57]
3-(3-pyridylmethylidene)-2-indolinone	DSS-induced C57BL/6J and ICR mice model of colitis	2.2, 11, and 22 mg/kg	↑DAI, ↓length of the colon, ↑infiltration of inflammatory cells, ↑loss of colonic crypts, and ↑epithelial cell necrosis	↑ IL-6 mRNA, ↑TNFα mRNA, ↑ IFN-γ mRNA,↑ NF-κB mRNA, ↑p65, ↑MCP-1, ↓Nrf2 activation and signaling, and ↓Nrf2 target genes activation and expression	↓NF-κB signaling and activation, ↓p65, ↓TNF-α mRNA, ↓IFN-y mRNA, ↓IL-6 mRNA, ↑Nrf2 activation and signaling, ↑NQO1 mRNA, ↑HO-1 mRNA and Nrf2 target genes activation and expression	[58]
Ruscogenins ** 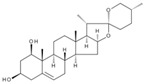 **	DSS-induced C57BL/6J mice model of colitis in vivo and LPS-induced inflammation in colonic organoids in vitro	0.5, 1, and 2 mg/kg in vivo and 50 μg/mL in vitro	↑DAI, ↑apoptosis, ↑intestinal barrier dysfunction, ↑inflammation in the intestinal epithelium, ↑bacterial translocation, ↑abscesses and ↑crypt ulcers in vivo and ↑inflammatory burden lesions in vitro	↑TNF-α, ↑ IFN-γ, ↑NLRP3, ↑caspase1, ↑caspase-11, ↑GSDMD, ↑apoptosis, ↑Bax, and ↑caspase-3 in vivo and ↑TNF-a, ↑IFN-y, ↑factors related to macrophage migration (G-CSF, RANTES, and MCP1), ↑apoptosis, ↑Bax, ↑caspase-3 expression, and ↓Bcl-2 in vitro	↑Nrf2/NQO1, ↑expression of proteins associated with the Nrf2/NQO1 pathway, ↑Bcl-2 protein and ↓Bax/c-caspase-3 signaling pathway in vivo and ↑Nrf2, ↑NQO1, ↓TNF-α, ↓IFN-γ, and ↓expression of factors related to macrophage migration in vitro	[59]
Caffeic acid ** 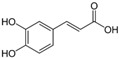 **	DSS-induced ICR mice model of colitis	251 mg/kg	↑DAI, ↑intestinal permeability and ↑intestinal infiltration, ↓goblet cells, ↑weight loss, ↓colon length, and ↑intestinal microbiota dysbiosis	↑IL-6, ↑TNF-α, ↑IL-1β, ↑IL-12, ↑MDA, ↑ROS, ↓ZO-1, ↓occludin, ↓IL-10, ↓GSH-Px, ↓SOD, and ↓ CAT	↓IL-1β mRNA, ↓IL-6 mRNA and ↓TNF-α mRNA, ↑SOD, ↑GPX1, ↑GPX2, ↑CAT, ↑IL-10 mRNA, ↑Nrf2 mRNA, ↑HO-1 mRNA, ↑NQO1, and ↑occludin mRNA	[60]
Schisandrin B ** 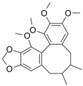 **	DSS-induced C57BL/6 mice model of colitis in vivo and HCT-116 cells induced by LPS in vitro	10 mg/kg in vivo and 40 uM in vitro	↑DAI, ↑intestinal ulceration, ↑intestinal permeability, ↑bacterial translocation, ↑immune cell infiltration, and ↓body weight in vivo and ↑intestinal inflammation and ↑intestinal OS in vitro	↑TNF-α, ↑IL-6, ↑IL-18, and ↑IL-1β in vivo and ↑TNF-α, ↑IL-6, ↑IL-18, ↑IL-1β, ↑NLRP3, ↑ ROS, ↑GSDMD protein, ↑LDH activity, ↑cellular apoptosis, and ↑mitochondrial damage in vitro	↑AMPK/Nrf2, ↑ NRF2, ↑ pAMPK, ↓ TNF-α, ↓ IL-6, ↓ IL-18, ↓ IL-1β, ↓ NLRP3 inflammasome, and ↓GSDMD protein expression in vivo and ↓TNF-α, ↓IL-6, ↓IL-18, ↓IL-1β, ↓NLRP3 protein expression, ↓NLRP3 activity, ↓GSDMD protein expression, ↑p-AMPK protein expression, and ↑Nrf2 activity in vitro	[61]
GB1a	DSS-induced C57BL/6 mice model of colitis	25, 50, and 100 mg/kg	Weight loss, ↑DAI, ↓number of crypts, and ↑infiltration of inflammatory cells in the mucosa and submucosa	↑MPO mRNA, ↑IL-6 mRNA, ↑TNF-α mRNA, ↑CCL5, ↑CCL20, ↑CXCL1, ↑NF-κBp65, ↑NF-κBp65 translocation to the nucleus, ↑MDA, ↓GSH, ↓SOD, ↓HO-1, ↓Nrf2 translocation to the nucleus and Nrf2-related proteins expression, ↓ZO-1 mRNA, and ↓occludin mRNA	↓MPO mRNA, ↓IL-6 mRNA, ↓TNF-α mRNA, ↓CCL5, ↓CCL20, ↓CXCL1, ↓NF-κBp65, ↓NF-κBp65 translocation to the nucleus, ↓MDA, ↑GSH, ↑SOD, ↑HO-1, ↑Nrf2 translocation to the nucleus and Nrf2-related proteins expression, ↑ZO-1 mRNA, and ↑occludin mRNA	[62]
Diosmetin ** 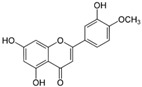 **	DSS-induced C57BL/6 mice model of colitis in vivo and Caco-2 and IEC-6 treated with LPS in vitro	25 and 50 mg/kg in vivo and 0–400 μM in vitro	↑DAI, ↓body weight, ↓colon length, ↑inflammatory infiltration, ↑intestinal permeability, and ↑intestinal epithelial barrier dysfunction in vivo and ↑intestinal inflammation and ↑intestinal OS in vitro	↑ROS, ↑IL-1β, ↑IL-6, ↑TNF-α, ↑COX-2, ↑MDA ↓occludin, ↓claudin-1, ↓GSH, ↓GSH-Px and ↓SOD in vivo and ↓occludin, ↓claudin and ↑ROS in vitro	↑GSH-Px, ↑SOD, ↑GSH, ↑Sirt1, ↑Nrf2 activation, ↑HO-1, ↑ZO-1, ↓MDA, ↓NF-κB signaling, ↓IL-1β mRNA, ↓IL-6 mRNA, and ↓COX-2 mRNA in vivo and ↓ROS, ↓NF-κB signaling, ↑claudin-1, ↑occludin, ↑Sirt1 expression, ↑Nrf2 expression and signaling, and ↑HO-1, ↑ZO-1 in vitro	[63]
Atractylenolide III ** 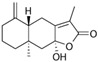 **	TNBS-induced C57BL/6 mice model of colitis	5, 10, or 20 mg/kg	↑DAI, ↑inflammation, ↑weight loss, ↓colon length, ↑inflammatory cell infiltration, ↑OS, ↑submucosal necrosis, ↑structural mucosal distortion, and ↑ulcerated areas	↑MPO, ↑IL-1β, ↑TNF-α, ↑MDA, ↑ROS, ↑FPR1, ↑NOX1, ↑phosphorylated (p)-Nrf2, ↓Nrf2 activation and signaling, ↓SOD, ↓GSHPx, and ↓CAT	↑Nrf2 activation and signaling, ↑CAT, ↑SOD, ↑GSH-Px, ↓mRNA of IL-1β and TNF-α, ↓MDA, ↓FPR1, ↓NOX1, ↓ (p)-Nrf2 phosphorylated, and ↓MPO	[64]
Polydatin ** 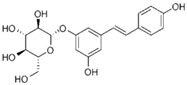 **	DSS-induced C57BL/6 mice model of colitis and LPS-treated RAW264.7 cells	Not reported in vivo and 100 μM, 200 μM, 300 μM and 400 μM in vitro	↑DAI, ↓colon length, ↑intestinal inflammation, and ↑intestinal OS in vivo and ↑inflammation and ↑OS in vitro	↑TNF-α, ↑IL-4, ↑IL-6, ↓IL-10, ↑JNK phosphorylation, and ↑COX-2	↑Nrf2 nuclear translocation and phosphorylation, ↓Erk1/2 phosphorylation, ↓JNK1/2 phosphorylation, ↑HO-1, ↑NQO1, ↑Akt phosphorylation, and ↑IL-10	[65]
	DNBS-induced CD1 mice model of colitis	10 mg/kg	↑Intestinal inflammation and ↑intestinal OS, ↑prominent transmural inflammatory cells infiltration, and ↑ulcer formation	↑IL-1β, ↑TNF-α, ↑prostaglandins, ↑NO, ↓HO-1, and ↑NF-κB-p65 phosphorylation	↓NF-κB translocation and ↓IkBα degradation, ↑SIRT1, ↑Nrf2, ↑HO-1, ↓pro-inflammatory genes expression, and ↓OS genes expression	[66]
Rosmarinic Acid ** 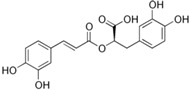 **	DSS-induced C57BL/6 mice model of colitis	5, 10, and 20 mg/kg	↑DAI, ↑intestinal inflammation, and ↑prominent transmural inflammatory cells infiltration	↑TNF-α, ↑IL-1β, ↑MPO, and ↑ inflammasome-related proteins	↑Nrf2 expression, ↑HO-1, ↓pro-inflammatory genes expression, and ↓OS genes expression	[67]
Imperatorin ** 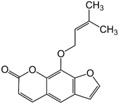 **	TNBS-induced SD mice model of colitis	15, 30, and 60 mg/kg	↑DAI, ↑intestinal inflammation, and ↑intestinal OS	↑ROS, ↑TNF-α and ↑IL-6	↑Nrf-2 expression, ↑ARE, ↑HO-1, and ↓OS genes expression	[68]
Berberine ** 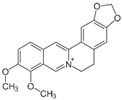 **	AcOH-induced Wistar mice model of colitis	25 and 50 mg/kg	↑Intestinal inflammation, ↑prominent transmural inflammatory cells infiltration, ↑mucosal edema, and ↑necrosis	↑IL-1β, ↑IL-6, ↑TNF-α, ↑MPO, and ↑PGE2	↑p38MAPK mRNA, ↓OS genetic expression, ↓apoptotic activity, and ↑Nrf2/HO-1 signaling pathway activity	[69]
Curcumin ** 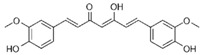 **	Hypoxia and hypothermia-induced NEC mice model of colitis	20 and 50 mg/kg	Intestinal inflammation	↑IL-1 β, ↑IL-6, ↑IL-18, and ↑TNF-α	↑SIRT1/NRF2 pathway, ↓TLR4 expression, and ↓pro-inflammatory genes expression	[70]
Sesamin ** 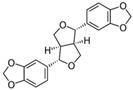 **	Caco-2 human intestinal epithelial cells exposed to H2O2	10, 20, 40, 80, 160, and 320 mM	↑Intestinal inflammation, ↑intestinal OS, and ↑intestinal cytotoxicity	↑ROS, ↓GSH, ↑IL-1β, ↑IL-6, ↑TNF-α, and ↑M1 macrophages polarization	↓Keap1, ↑Nrf-2/ARE expression, ↑p-AKT/AKT, ↑GCLC, ↑GCLM, ↑NQO1, and ↑HO-1	[71]
Toosendanin ** 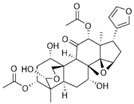 **	DSS-induced C57BL/6 mice model of colitis	0.5 mg/kg and 1 mg/kg	↑DAI, ↑intestinal inflammation, and ↑intestinal OS	↑IL-1β, ↑IL-6, ↑TNF-α, ↑RONS, and ↓GSH	↑Nrf2 expression, ↑HO-1, ↓pro-inflammatory genes expression, and ↓OS genes expression	[72]
Galangin ** 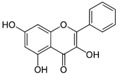 **	LPS-treated RAW264.7 cells and DSS-induced Balb/c mice model of colitis	0.39 and 0.78 µg/mL for LPS-treated cells and 20 and 40 mg/kg for mice	↑DAI, ↑loss of epithelial and goblet cells, ↑crypt aberrations in vivo and ↑inflammation and ↑OS in vitro	↑Nitrites, ↑IL-6, ↑TNF-α, ↓SOD, ↓GSH, ↑NF-κB activation, ↑iNOS, and ↑COX-2	↑Nrf2 signaling pathway activation, ↑HO-1, ↓pro-inflammatory genetic expression, ↓JNK phosphorylation, and ↓p-IKKα/β	[73]
Apocynin ** 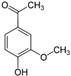 **	DSS-induced Balb/c mice model of colitis	400 mg/kg	↑Intestinal inflammation and ↑intestinal OS	↑iNOS, ↑COX-2, ↑TNF-α, ↑IL-1β, ↑IL-6, and ↑MCP-1	↑Nrf2 signaling pathway activation, ↑HO-1, and ↓pro-inflammatory genetic expression expression	[74]
Hesperidin ** 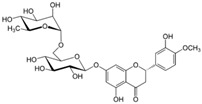 **	DSS-induced C57BL/6 mice model of colitis and TNF-α/IFN-γ treated Caco-2 cells	10, 20, or 40 mg/kg in vivo and 10, 20 or 40 μg/mL in vitro	↑Intestinal inflammation	↑TNF-α, ↑IL-6, ↑IL-10, and ↑MPO	↑Nrf2 antioxidant signaling pathways activation, ↑NQO1, ↑HO-1, ↑regulatory T cells expression, ↓MPO ↓TNF-α expression, ↓IL-6 expression, and ↑IL-10 expression	[75]
Norisoboldine ** 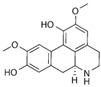 **	TNBS-induced Balb/c rats model of colitis	20 and 40 mg/kg	↑Intestinal inflammation and ↑intestinal OS	↑TNF-α, ↑IL-1β, and ↑IL-6	↓NLRP3 inflammasome, ↑Nrf2 signaling pathway activation, and ↑Nrf2 expression	[76]
Hyperoside ** 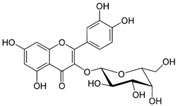 **	DSS-induced C57BL/6 mice model of colitis	80 and 120 mg/kg	↑DAI, ↑intestinal inflammation ↑intestinal OS, and ↓colon length	↑TNFα, ↑IL-4, ↑IL-6, and ↑ROS	↑Nrf2 signaling pathway activation, ↑HO-1, ↑SOD mRNA, ↓pro-inflammatory genetic expression, and ↓NF-κB expression	[77]
Glyceollins 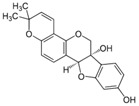 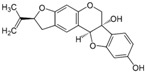	DSS-induced Balb/c rats model of colitis	4 and 10 mg/kg	↑DAI, ↑intestinal inflammation, ↑intestinal OS, ↑prominent transmural inflammatory cells infiltration, and ↑mucosal necrosis	↑TNFα, ↑IL-6, ↑iNOS, and ↑COX-2	↓NF-κB expression, ↑Nrf2 signaling pathway activation, and ↓pro-inflammatory genetic expression	[78]
Carnosic acid 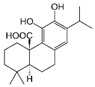	DSS-induced mice model of colitis	50 and 100mg/kg	↑DAI, ↑intestinal inflammation, ↑intestinal OS, and ↑prominent transmural inflammatory cells infiltration	↑TNF-α, ↑IL-17A, ↑IL-6, ↑IFN-γ, ↑IL-1β, ↑IL-18, and ↑NF-κB activation	↑Nrf2 expression, ↓Nrf2 ubiquitination and degradation, ↓NLRP3 inflammasome, ↑GCLM, ↑NQO1, ↑HO-1, ↓OS genes expression, and ↓Keap1	[79]
Protocatechuic acid ** 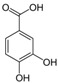 **	TNBS-induced Balb/c mice model of colitis	30 and 60 mg/kg	↑Intestinal inflammation and ↑intestinal OS	↑IL-6, ↑TNF-α, ↑IL-1β, and ↑COX-2	↑Nrf2 expression, ↓COX-2 expression, and ↓NF-κB expression	[80]

↑, increase; ↓, decrease; AcOH, acetic acid; AKT, advanced protein kinase B; ARE, antioxidant response element; ARKs, aldo-keto reductases; COX-2, cyclooxygenase; DAI, disease activity index; DSS, dextran sulfate sodium; DNBS, dinitrobenzene sulfonic acid; Erk, extracellular signal-regulated kinases; FPR1, Formyl peptide receptor 1; GCLC, catalytic glutamate-cysteine ligase; GCLM, glutamate-cysteine ligase modifier subunit; GPX1, glutathione peroxidase 1; GPX2, glutathione peroxidase 2; GR, glutathione reductase; GSH, glutathione; GSH, glutathione; HO-1, heme oxygenase 1; IL-1β, interleukin 1 beta; IL-6, interleukin 6; IL-10, interleukin 10; iNOS, inducible nitric oxide synthase; JNK, c-Jun N-terminal kinase; Keap1, Kelch-like ECH associated protein 1; LDH, lactate dehydrogenase; LPS, lipopolysaccharide; MAPK, mitogen-activated protein kinase; MCP-1, monocyte chemoattractant protein-1; mTOR, mammalian target of rapamycin; mRNA, messenger RNA; MPO, myeloperoxidase; MUC2, mucin 2, oligomeric mucus gel-forming; NEC, necrotizing enterocolitis; NF-κB, nuclear factor kappa b; NF-κB-p65, NF-κB transcription factor p65; NLRP3, NLR [nucleotide-binding domain and leucine-rich repeat containing] family pyrin domain containing 3; NO, nitric oxide; NOX1, NADPH (nicotinamide adenine dinucleotide phosphate hydrogen) oxidase 1; NQO1, quinone oxidoreductase; Nrf2, nuclear factor erythroid 2-related factor 2; PCG-1, polycomb-group proteins 1; PGE2, prostaglandin E2; p-IKKα/β, IKK serine kinase proteins α/β; RONS, reactive oxygen and nitrogen species; ROS, reactive oxygen species; SD, Sprague Dawley; SIRT1, silent information regulator 2-related protein 1; SOD, superoxide dismutase; SW480 cells, human intestinal epithelial cell lines; TFAM, mitochondrial transcription factor A; TLR4, toll-like receptor; TNBS, 2,4,6-trinitro-benzenesulfonic acid; TNF-α, tumor factor necrosis; UDP-GT, uridine 5’-diphospho-glucuronosyltransferase.
